# Ageing restructures the transcriptome of the hypothalamic supraoptic nucleus and alters the response to dehydration

**DOI:** 10.1038/s41514-023-00108-2

**Published:** 2023-06-01

**Authors:** Ghadir Elsamad, André Souza Mecawi, Audrys G. Pauža, Benjamin Gillard, Alex Paterson, Victor J. Duque, Olivera Šarenac, Nina Japundžić Žigon, Mingkwan Greenwood, Michael P. Greenwood, David Murphy

**Affiliations:** 1 Molecular Neuroendocrinology Research Group, Bristol Medical School: Translational Health Sciences, Dorothy Hodgkin Building, University of Bristol, Bristol, England; 2 Laboratory of Molecular Neuroendocrinology, Department of Biophysics, Paulista School of Medicine, Federal University of São Paulo, São Paulo, Brazil; 3 Insilico Consulting Ltd., Wapping Wharf, Bristol, England; 4 Institute of Pharmacology, Clinical Pharmacology and Toxicology, Faculty of Medicine, University of Belgrade, Belgrade, Serbia; 5 Present Address: Translational Cardio-Respiratory Research Group, Department of Physiology, Faculty of Medical and Health Sciences, University of Auckland, Auckland, New Zealand; 6 Present Address: Department of Safety Pharmacology, Abbvie, North Chicago, Illinois USA

**Keywords:** Ageing, Stress and resilience

## Abstract

Ageing is associated with altered neuroendocrine function. In the context of the hypothalamic supraoptic nucleus, which makes the antidiuretic hormone vasopressin, ageing alters acute responses to hyperosmotic cues, rendering the elderly more susceptible to dehydration. Chronically, vasopressin has been associated with numerous diseases of old age, including type 2 diabetes and metabolic syndrome. Bulk RNAseq transcriptome analysis has been used to catalogue the polyadenylated supraoptic nucleus transcriptomes of adult (3 months) and aged (18 months) rats in basal euhydrated and stimulated dehydrated conditions. Gene ontology and Weighted Correlation Network Analysis revealed that ageing is associated with alterations in the expression of extracellular matrix genes. Interestingly, whilst the transcriptomic response to dehydration is overall blunted in aged animals compared to adults, there is a specific enrichment of differentially expressed genes related to neurodegenerative processes in the aged cohort, suggesting that dehydration itself may provoke degenerative consequences in aged rats.

## Introduction

Ageing is accompanied by an increased prevalence of disorders of body salt and water composition. As revealed by the UK Dehydration Recognition In Our Elders (DRIE), 20% of residents in care are dehydrated^1^. Many elderly patients admitted to the hospital present osmotic balance disorders, and dehydration (DH) is often a cause of morbidity and mortality in senior citizens^2–4^. Hypo/hypernatremia are much more prevalent in the elderly and are associated with falls, fractures and osteoporosis^2,4^. Thus, to improve healthy living among the elderly, we need to understand why salt and water imbalances occur in this age group. Both peripheral and central mechanisms controlling salt and water homoeostasis change with age. Ageing is accompanied by a gradual decline in renal function, with urine-concentrating capacities reduced in the elderly compared to younger subjects^5^. This diminished ability to conserve bodily water, accompanied by reduced thirst and insufficient water intake after fluid deprivation, makes the elderly more prone to DH^6,7^.

The neuroendocrine reflexes regulating hydromineral balance are centred on the large peptidergic magnocellular neurones (MCNs) mainly located in the supraoptic nucleus (SON), the core hypothalamic osmoregulatory centre^8,9^. Distinct groups of SON MCNs produce two major neuropeptide hormones, arginine vasopressin (AVP; encoded by the *Avp* gene) and oxytocin (OXT; encoded by the *Oxt* gene)^10^, which are stored in its synaptic terminals located at the neurohypophysis. The rise in plasma osmolality consequential to DH is detected by independent MCN mechanisms^11,12^ and by osmoreceptive neurones in the circumventricular organs, such as the subfornical organ, which project to MCNs and whose excitatory inputs trigger the firing activity of MCNs to elicit hormone secretion^13^. Upon release, AVP and OXT travel through the circulation to control water and sodium reabsorption in the kidney^14–16^, contributing to body water retention and sodium excretion (8.9). Chronic osmotic stimulation depletes pituitary stores of AVP; thus, there is a need to synthesise more AVP. This is initiated by augmented *Avp* gene transcription^17^, which leads to a concomitant increase in the levels of the *Avp* precursor heteronuclear RNA^18^ and the processed cytosolic mature mRNA^19^.

We have shown that aged rats drink less than their younger counterparts and exhibit higher plasma osmolality under both euhydrated (EH) and DH conditions^20^. Following DH, aged animals consume less water and less salt^21^. Pituitary vasopressin content is high in older animals under DH conditions, implying reduced secretion^20^. AVP gene transcription (indirectly measured by quantifying AVP hnRNA by quantitative reverse transcriptase PCR, qRT-PCR) is increased in old age in the basal state^20^. These physiological and molecular data suggest that excitation-synthesis-secretion coupling in the SON^22^ is altered in old age.

Latterly it has become evident that hydration status is connected to metabolic health. People who drink less water have an increased risk of developing type 2 diabetes, and this seems to be associated with elevated AVP levels^23^. Sundry studies have found that increased AVP release (commonly assessed by quantification of the surrogate precursor product copeptin) is associated with insulin resistance, type 2 diabetes and major cardiovascular events that characterise the metabolic syndrome^24–27^. Further, new evidence supports a causative role for long-term increases in circulating AVP in the development of many morbidities of old age^28–34^.

In contrast to the physiological alterations evident during ageing, the anatomical structure of the SON is remarkably stable with age and resistant to deterioration consequential to Alzheimer’s disease (AD). Studies on the aged human brain have revealed maintenance of the structure of the SON cytoskeleton and MCN activity and connectivity^35–40^. The characteristic morphological features of AD, amyloid ß (Aß)-positive plaques and tau-positive neurofibrillary tangles (NFTs), are rarely seen in the SON^41–45^.

A striking functional remodelling of the HNS results from chronic osmotic stimulation^46,47^. Changes in the morphology, electrical properties and biosynthetic and secretory activity of the SON have been documented^48^, all of which are thought to promote hormone production and secretion. We sought to describe the transcriptomic basis of this function-related plasticity. Thus, we have used Affymetrix GeneChip^49–51^, and latterly RNASeq^52,53^, to comprehensively catalogue the transcriptomes of the young adult male and female rat SON and to document how these are changed by the chronic osmotic challenges of DH or salt loading. We have carried out a detailed functional investigation of many of these differentially expressed genes; for example, *Creb3l1*^54^. These studies have established the SON as a tractable model that facilitates the transition from extensive transcriptome datasets to new physiological knowledge^55^.

In this report, we describe the use of Illumina RNAseq to comprehensively catalogue the polyadenylated transcriptomes of the Wistar Han rat SON from 18-month-old euhydrated and dehydrated rats. Comparison of the new catalogues with our previously published datasets from 3-month-old EH and DH rats^53^ has enabled us to identify the changes in gene expression that result from ageing, as well as common and age-specific responses to DH. These datasets are a valuable resource for continued studies on the ageing SON, which may we have implications for the health and well-being of the elderly.

## Results

We have asked about the process of ageing in the Wistar Han supraoptic nucleus (SON). We compared adult (3-month-old) and aged (18-month-old) animals. Physiological assessment of the ageing process in these rats was performed and revealed that aged rats had a significantly higher weight than adult rats, which, in both adult and aged animals, was significantly decreased by DH (Supplementary Fig. 1a). Whilst rats in both age groups showed a significant increase in plasma osmolality and plasma AVP following DH (Supplementary Fig. 1a), both basal and dehydrated plasma osmolality were elevated in aged animals compared to adults (Supplementary Fig. 1a).

We have previously sequenced the SON polyadenylated transcriptome from EH (*n* = 5) and DH (*n* = 5) adult rats. Applying a *P*_adj_ value cut-off of 0.05 (with Benjamini–Hochberg correction) with no log2 fold change (LFC) filter, a total of 2246 differentially expressed (DEGs) were identified^53^. Here we report the sequencing of the SON polyadenylated transcriptome from EH (*n* = 5) and DH (*n* = 5) aged rats. RNASeq samples from the four groups (adult EH, adult DH, aged EH, aged DH) clustered separately, illustrating transcriptome differences between the experimental conditions (Supplementary Fig. 1b). Using the same statistical and filtering parameters as for the adult samples, we have identified genes that change in expression as a consequence of ageing (1283 genes) and genes that alter their expression following DH in aged rats (1536 genes). Our new ageing-related transcriptomic datasets are presented in Supplementary Data 1 and are publically accessible (https://www.ncbi.nlm.nih.gov/geo/query/acc.cgi?acc=GSE214353). Several identified DEGs were validated using quantitative reverse transcription PCR (qRT-PCR) in SON samples from an independent cohort of adult (EH and DH) (Supplementary Fig. 1c) and aged (EH and DH) animals (Supplementary Fig. 1d), indicating a low false discovery rate in our findings. Note that LogFC of the RNAseq and qRT-PCR expression are closely correlated (Supplementary Fig. 1e, f).

### Weighted Correlation Network Analysis of SON transcriptomes and physiological parameters

We used Weighted Correlation Network Analysis (WGCNA)^56^ to tease physiological meaning from our transcriptome datasets. WGCNA uses correlations of gene expression patterns to define modules of closely related genes, with modules assigned arbitrary colour names. Genes within modules are assumed to have some functional overlap. Here, we used WGCNA to define groups of genes that may share functional pathways in response to dehydration that are also influenced by ageing and identify influential “hub” genes within these modules that may be promising candidates for further investigation. Modules of genes identified by expression can then be correlated with physiological trait data. We used weight as a proxy for the age of the animals and plasma osmolality and plasma AVP as markers for the dehydration response. WGCNA is an especially useful method when there are more than two groups to compare. Therefore, all four groups (EH adult, EH aged, DH adult and DH aged) were used in this WGCNA analysis. Eigengenes (an abstract representation of the genes in a module) for each module identified by WGCNA were correlated (bicor) to trait data and the categorical groups of adult/aged and EH/DH (Supplementary Fig. 1g).

### Ageing induced transcriptome dynamics in the rat SON

**Fig. 1:**
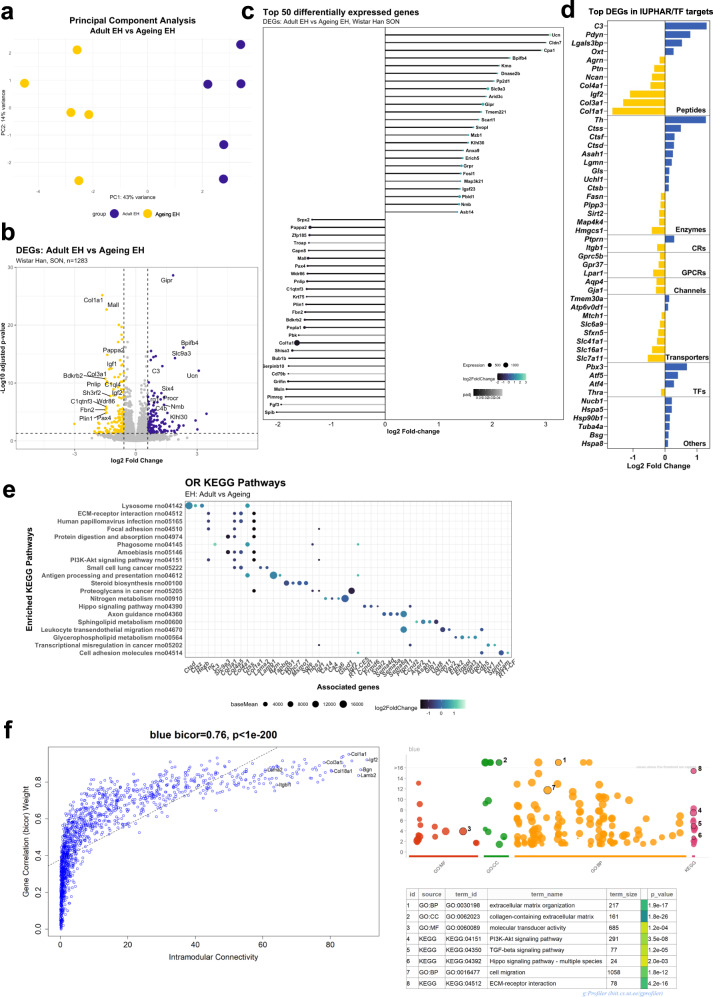
Transcriptome dynamics of the ageing rat SON. PCA plot illustrating distinct differences between the adult and ageing SON (**a**). Volcano plot showing the distribution of DEGs (**b**). Top 50 ageing DEGs (**c**). Classification of top 50 DEGs; CRs catalytic receptors, GPCRs G protein-coupled receptors, TFs transcription factors (**d**). Dot plot showing pathway analysis against the KEGG database and their associated DEGs. The top five most significant DEGs by *P*_adj_ associated with each over-represented KEGG term are shown as dots on the *x*-axis; colour denotes the direction of changed expression (LFC), and size denotes expression level. Enriched KEGG terms are plotted on the *y*-axis. Expression is colour-coded (**e**). WGCNA analysis showing the “blue” module and its associated enriched GO pathways (MF, BP, CC and KEGG) (**f**).

In order to identify DEGs associated with ageing in the rat SON, we compared the adult EH and the aged EH datasets. These datasets clustered separately in a PCA plot (Fig. 1a). Of the 1283 DEGs associated with ageing in the SON, 586 were upregulated, and 697 were downregulated. To help visualise the data intuitively, a volcano plot was used (Fig. 1b). Note that all gene symbols referred to in this manuscript are defined in Supplementary Table 1.

We categorised the global gene expression profile of the ageing SON, firstly by plotting the top 50 DEGs (*P*_adj_ < 0.05) ordered by LFC in a lollipop graph that also plots significance (by *P*_adj_ value) and expression level represented by the size of the dot (Fig. 1c). DEGs were then catalogued according to their identity as transcription factors^57^ or their physiological or pharmacological classifications using the International Union of Basic and Clinical Pharmacology and British Pharmacological Society (IUPHAR- BPS; ^58^) (Fig. 1d). The full classification list can be found in Supplementary Data 2.

To assist with the biological understanding of the ageing data, Gene Ontology (GO; ^59^), Reactome^60^ and KEGG^61^ analyses were performed on the ageing DEG set. The DEGs list was used without any filtering being applied to the LFC nor the basemean counts. Eighteen Molecular Function (MF) GO enriched terms were identified to be significantly over-represented in adult versus aged rat SON. Cell adhesion (GO:0050839; *P*_adj_ = 1.17E-7), extracellular matrix (ECM) structure (GO:0005201; *P*_adj_ = 1.17E-7), and integrin binding (GO:0005178; *P*_adj_ = 1.22E-6) were the main enriched molecular function terms (Supplementary Fig. 1h, Supplementary Data 3).

Over-representation analysis (ORA) using the Reactome databases (Supplementary Fig. 1i, Supplementary Data 5) identified extracellular matrix organisation (R-RNO-1474244; *P*_adj_ = 6.61E-9) as the most over-represented Reactome pathway, which was also present in the MF GO analysis. Multiple pathways connected to the ECM were highly represented in this analysis, such as collagen formation (R-RNO-1474290; *P*_adj_ = 3.6E-4), ECM proteoglycans (R-RNO-3000178; *P*_adj_ = 2.8E-4), laminin interaction (R-RNO-3000157; *P*_adj_ = 6.6E-4), and collagen degradation (R-RNO-1442490; *P*_adj_ = 2.9E-3).

Ageing of the SON was defined by 20 over-represented KEGG pathways. The analysis confirmed the enrichment of the ECM pathways, including lysosomes (rno04142; *P*_adj_ = 1.96E-8) and ECM-receptor interaction (rno04512; *P*_adj_ = 3.14E-8) (Fig. 1e, Supplementary Data 5).

Considering the WGCNA analysis, the “blue module” showed a strong negative correlation with weight (bicor −0.9, *P* ≤ 0.001) but little correlation with plasma AVP or plasma osmolality (Supplementary Fig. 1e), suggesting it represents genes influenced specifically by ageing. GO analysis using genes assigned to the ‘blue module’ corroborated ORA results described above, with some structural terms such as extracellular matrix organisation (GO:0030198, *P*_adj_ = 1.9E-17) and collagen-containing extracellular matrix (GO:0062023, *P*_adj_ = 1.8E-25). The “blue module” showed a very strong correlation between within-module connectivity and correlation to the weight trait (bicor 0.76, *P* < 1e-200). The gene with the highest within-module connectivity was *Igf2*, which is involved in cell proliferation and growth. Other high connectivity genes in the “blue module” include the gene encoding the extracellular proteoglycan *Bgn* and the collagen encoding genes *Col4a5* and *Col1a1* (Fig. 1f).

### DH induced transcriptome dynamics in the aged rat SON

**Fig. 2:**
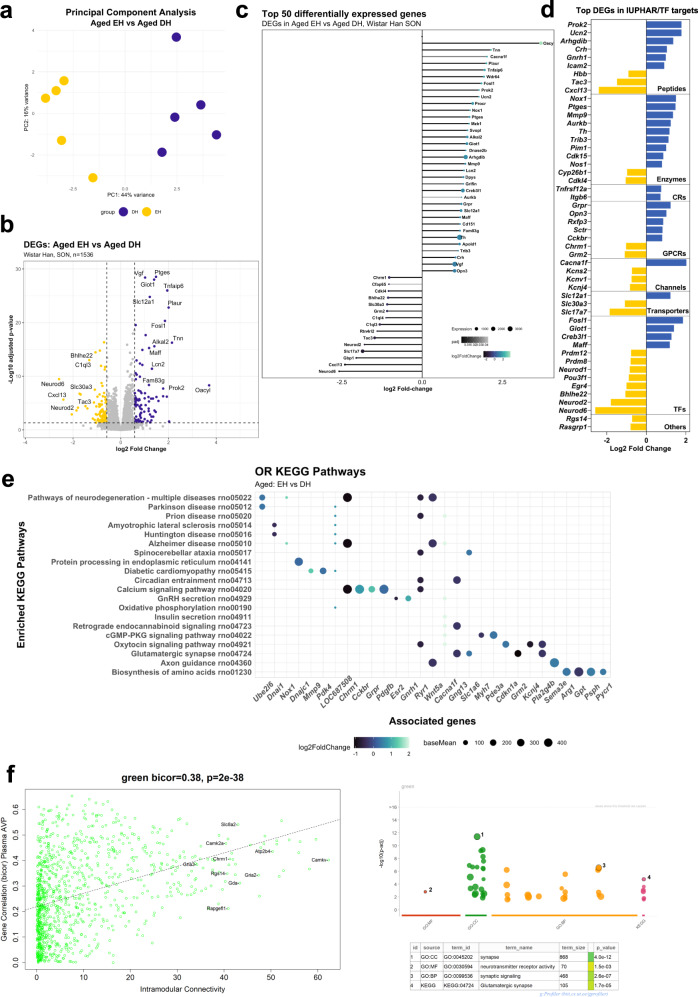
Transcriptome dynamics evoked by DH in the aged rat SON. PCA plot illustrating differences between the aged EH and DH groups (**a**). Volcano plot showing the distribution of DEGs (**b**). Top 50 aged DH DEGs (**c**). Classification of to 50 DEGs by LFC; CRs catalytic receptors, GPCRs G protein-coupled receptors, TFs transcription factors (**d**). ORA KEGG pathway analysis of the DEGs (**e**). WGCNA analysis showing the “green” module and its associated enriched GO pathways (MF, BP, CC and KEGG) (**f**).

In order to identify DEGs associated with DH in the aged rat SON, we compared the aged EH and the aged DH datasets. These datasets clustered separately in a PCA plot (Fig. 2a). Of the 1536 DEGs associated with DH in the aged SON, 842 were upregulated, and 694 were downregulated. To help visualise the data intuitively, a volcano plot was used (Fig. 2b). The top 50 DEGs are presented organised according to LFC in a lollipop graph (Fig. 2c). DEGs were then catalogued according to their identity as transcription factors or their physiological or pharmacological classifications (Supplementary Data 6) (Fig. 2d). ORA KEGG analysis of the DEGs revealed 55 enriched pathways including those involved in neurodegeneration (rno05022; *P*_adj_ = 5.9E-23), Parkinson’s disease (rno05012; *P*_adj_ = 2.6E-22), prion disease (rno05020; *P*_adj_ = 1.3E-19), and Alzheimer’s disease (rno05010; *P*_adj_ = 6E-16) (Fig. 2e, Supplementary Data 7).

Considering our WGCNA analysis, the “green module” is representative of DEGs in DH, specifically with correlation to Plasma AVP (bicor −0.8, <0.001; Supplementary Fig. 1e). This module was enriched for GO terms and KEGG pathway relating to synaptic transmission and activity (Fig. 2f). *CamkV*, which encodes a pseudokinase which is expressed at the synapse^62^, alongside other genes expressed in the synapse, reside within the “green module” with high connectivity and links to neuronal development genes such as *Chrm1 and Gria2*.

### Comparison of ageing DEGs with adult DH DEGs

**Fig. 3:**
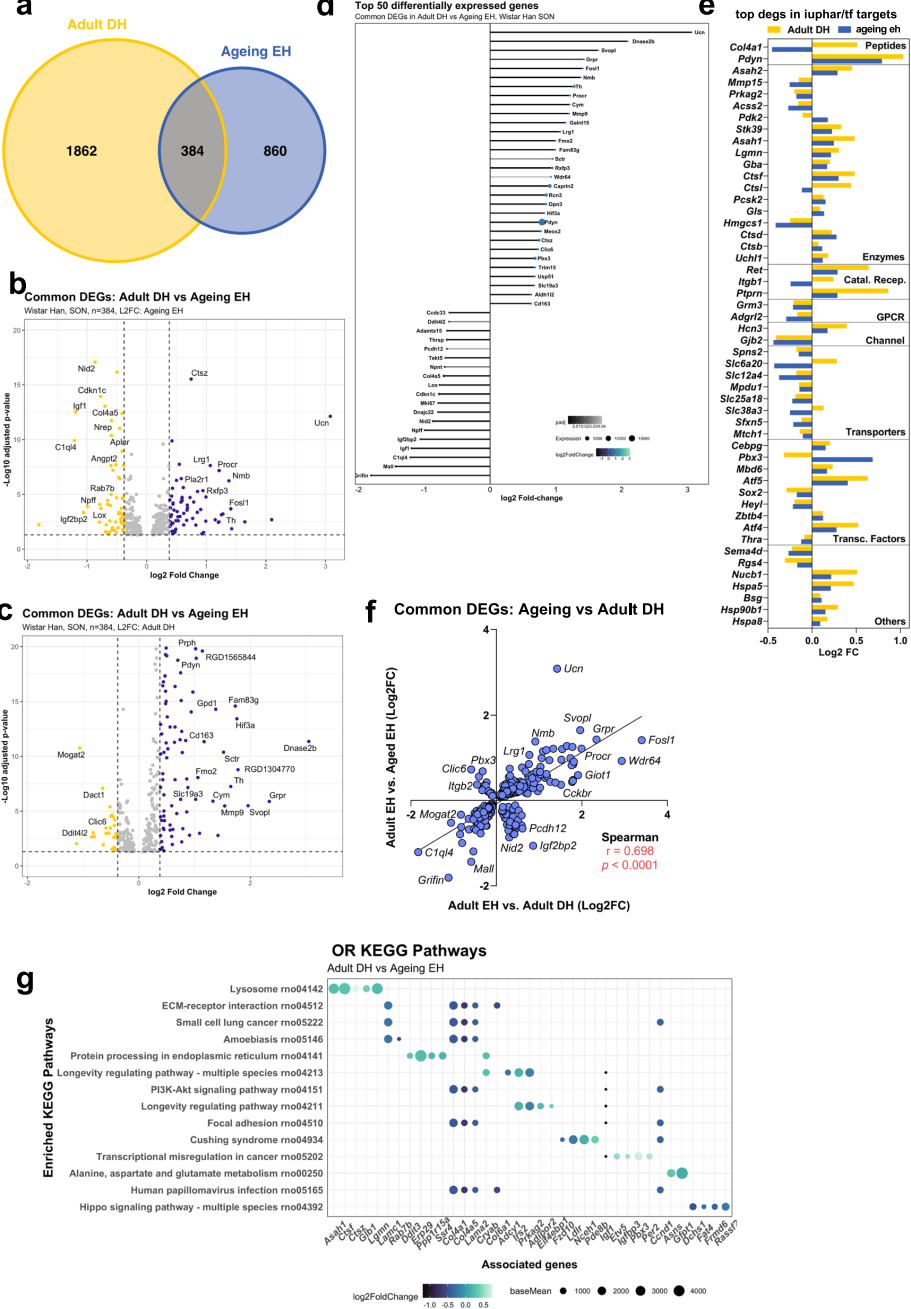
Comparison of adult DH DEGs with ageing DEGs. Venn comparison of adult DH and ageing DEGs (**a**). Volcano plot showing the distribution of DEGs using LFC of the ageing DEGs (**b**) or using LFC of the adult DH DEGs (**c**). Lollipop graph highlighting the top 50 DEGs common between the two datasets (**d**). Classification of the top 50 DEGs by LFC; Catal. Recep catalytic receptors, GPCR G protein-coupled receptor, Transc. Factors transcription factors (**e**). Correlation analysis (**f**) and ORA KEGG pathway analysis of the DEGs (**g**).

In order to identify genes regulated by dehydration in adult rats that are also sensitive to ageing, we compared the list of genes significantly modulated by ageing in the SON (1283, Fig. 1) to our previously published list of genes regulated by dehydration in the SON of adult rats (2246)^53^. This comparison revealed 384 DEGs commonly regulated by dehydration in adult rats and by ageing in the SON (Fig. 3a). These genes are displayed in a volcano plot using the LFC of the ageing EH dataset (Fig. 3b) and the adult DH dataset (Fig. 3c). The top 50 DEGs are presented organised according to LFC during ageing in a lollipop graph (Fig. 3d). DEGs were then catalogued according to their identity as transcription factors or their physiological or pharmacological classifications (Supplementary Data 8) (Fig. 3e). There is a positive correlation between the common DEGs of the two datasets (Fig. 3f). However, some genes upregulated by dehydration in the adult are downregulated by ageing (for example, *Igfbp2*, *Nid2* and *Pcdh12*). Conversely, some genes downregulated by dehydration in the adult are upregulated during ageing (for example, *Clic6*, *Itgb2* and *Pbx3*). ORA by KEGG of the common genes revealed pathways such as lysosomes (rno04142; *P*_adj_ = 1.86E-3), ECM-receptor interaction (rno04512; *P*_adj_ = 3.2E-3), protein processing in the endoplasmic reticulum (rno04141; *P*_adj_ = 1.9E-2), and longevity regulating pathway (rno04211; *P*_adj_ = 2.1E-2) (Fig. 3g, Supplementary Data 9).

### Comparison of ageing DEGs with aged DH DEGs

**Fig. 4:**
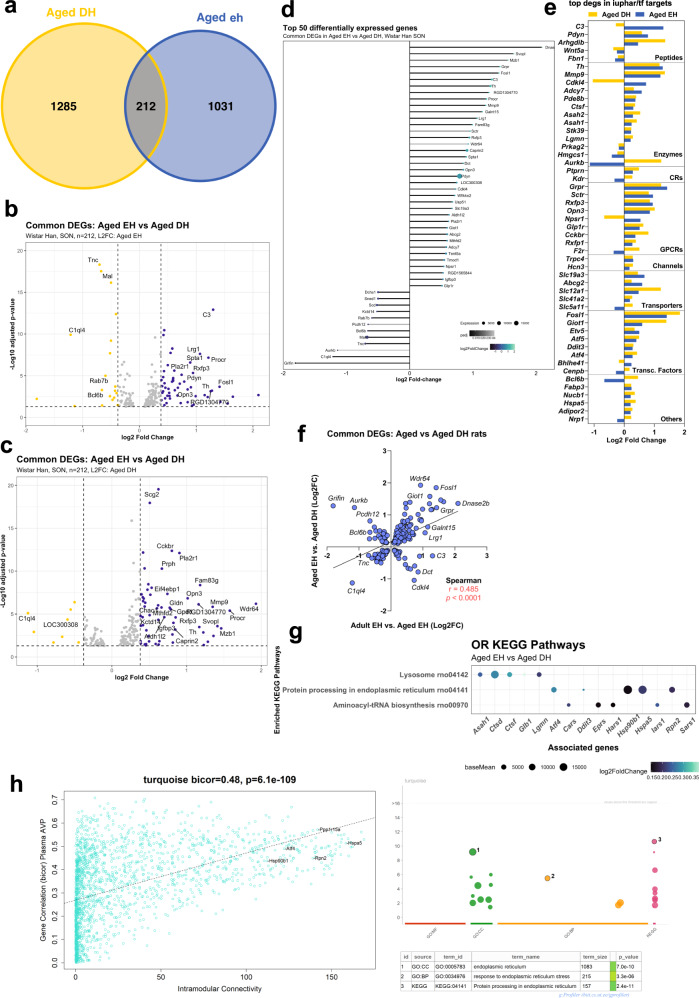
Comparison of aged DEGs with aged DH DEGs. Venn comparison of aged DH and ageing DEGs (**a**). Volcano plot showing the distribution of the DEGs using LFC of the ageing DEGs (**b**) or using LFC of the aged DH DEGs (**c**). Lollipop graph highlighting the top 50 DEGs common between the two datasets (**d**). Classification of the top 50 DEGS by LFC; CRs catalytic receptors, GPCRs G protein-coupled receptors, Transc. Factors transcription factors (**e**). Correlational analysis of the common DEGs (**f**). ORA KEGG pathway analysis of the common DEGs (**g**). WGCNA analysis showing the turquoise module and its associated enriched GO pathways (MF, BP, CC and KEGG) (**h**).

We found that 212 genes change in expression as a consequence of both ageing and DH in aged rats (Fig. 4a). These genes are displayed in a volcano plot using LFC of the ageing dataset (Fig. 4b). Different genes were highlighted when the common genes were displayed in a volcano plot using the LFC of the aged DH dataset (Fig. 4c). The top 50 DEGs are presented organised according to LFC during ageing in a lollipop graph (Fig. 4d). DEGs were then catalogued according to their identity as transcription factors or their physiological or pharmacological classifications (Supplementary Data 10) (Fig. 4e). There is a positive correlation between the common DEGs of the two datasets (Fig. 4f). However, some genes upregulated by dehydration in aged animals are downregulated by ageing (for example, *C3*, *Dct* and *Cdkl4*). Conversely, some genes downregulated by dehydration in the adult are upregulated during ageing (for example, *Tnc*, *C1ql4*). ORA KEGG analysis of the common genes revealed three enriched pathways: lysosomes (rno04142; *P*_adj_ = 1.7E-4), protein processing in the endoplasmic reticulum (rno04141; *P*_adj_ = 2E-2), and aminoacyl-tRNA biosynthesis (rno00970; *P*_adj_ = 2E-2) (Fig. 4g). DEGs that overlapped the aged DH and ageing comparisons were best represented by the “turquoise” WGCNA module (including 25% of module genes). The ‘turquoise” module, which was the largest cluster of genes identified by WGCNA, was enriched for terms involved in the protein folding process. Terms such as endoplasmic reticulum (GO:0005783; *P*_adj_ 7.00e-10), response to endoplasmic reticulum stress (GO:0034976; *P*_adj_ 3.275e-06), and protein processing in the endoplasmic reticulum (KEGG:04141; *P*_adj_ 2.412e-11) were all significantly enriched (Fig. 4h). Genes with high connectivity in the “turquoise” module included *Atf4* and *Hspa5* which are involved in protein folding processes and ER stress pathways^63,64^. Reassuringly, WGCNA analysis further reiterated the conclusions of the over-representation analyses. Our analysis suggests that stimulating the aged SON with DH aggravates the ageing process, and the SON attempts to cope with these stresses by activation of the unfolded protein response pathway.

### Comparison of adult DH DEGs with aged DH DEGs

**Fig. 5:**
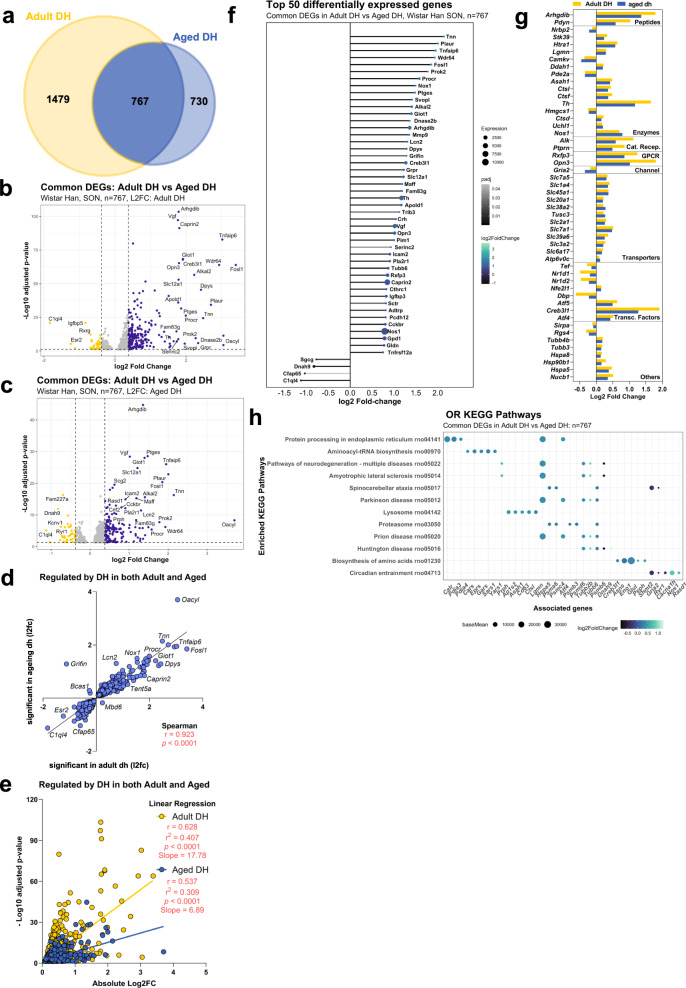
Comparison of adult DH DEGs with aged DH DEGs: common DEGs. Venn comparison of adult DH and aged DH DEGs highlighting the common genes (*n* = 767) (**a**). Volcano plot showing the distribution of the common adult DH and aged DH DEGs using LFC of the adult DH DEGs (**b**) or using LFC of the aged DH DEGs (**c**). Correlation analysis (**d**) and linear regression analysis (**e**) comparing the adult and aged responses to DH (**e**). Lollipop graph highlighting the top 50 DEGs common between the two datasets (**f**). Classification of the top 50 DEGS by LFC; Cat. Recep catalytic receptors, GPCR G protein-coupled receptor, Transc. Factors transcription factors (**g**). ORA KEGG pathway analysis of the common DEGs (**h**).

Comparison of adult DH significant DEGs with aged DH significant DEGs revealed that 767 genes change in expression as a consequence of dehydration in both adult and aged rats, 730 genes change only in aged DH, and 1479 genes change only in adult DH (Fig. 5a). Each of these gene categories will be considered separately.

#### Common DH DEGs

We found that 767 genes change in expression as a consequence DH in both adult and aged rats (Fig. 5a). These genes are displayed in a volcano plot using LFC of the adult (Fig. 5b) or aged (Fig. 5c) DH datasets. There was a strong positive correlation between aged and adult DH DEGs of the two datasets (Fig. 5d). Only one gene upregulated by dehydration in adult animals was downregulated in aged rats (*Mdb6*). Conversely, only three genes downregulated by dehydration in the adult were upregulated in DH aged animals (*Grifin*, *Bcas1* and *Qdpr*). Linear regression analysis comparing the common adult DH genes and aged DH DEGs (Fig. 5e) revealed that ageing decreased the *r*^2^ value from 0.407 to 0.309 and revealed a significantly smaller slope with aged compared to adult rats (*F* = 119.6, *P* < 0.0001). Further, comparing average absolute log2FC values, we found that aged rats have a much smaller SON transcriptomic response to WD (0.343) when compared to adult rats (0.405), at least for these 784 commonly DEGs (*P* < 0.0001 by Wilcoxon matched-pairs signed-rank test). Overall, these data suggest that the response to DH in old animals is blunted compared to adult animals.

The top 50 DEGs are presented organised according to aged DH LFC in a lollipop graph (Fig. 5f). DEGs were then catalogued according to their identity as transcription factors or their physiological or pharmacological classifications (Supplementary Data 11) (Fig. 5g). Pathways such as protein processing in the endoplasmic reticulum (rno04141; *P*_adj_ = 2.7E-11), aminoacyl-tRNA biosynthesis (rno00970; *P*_adj_ = 2.9E-07), and circadian entrainment pathway (rno04713; *P*_adj_ = 2.4E-2) (Fig. 5h, Supplementary Data 12) were amongst the enriched terms in ORA KEGG analysis.

#### Unique adult DH DEGS

**Fig. 6:**
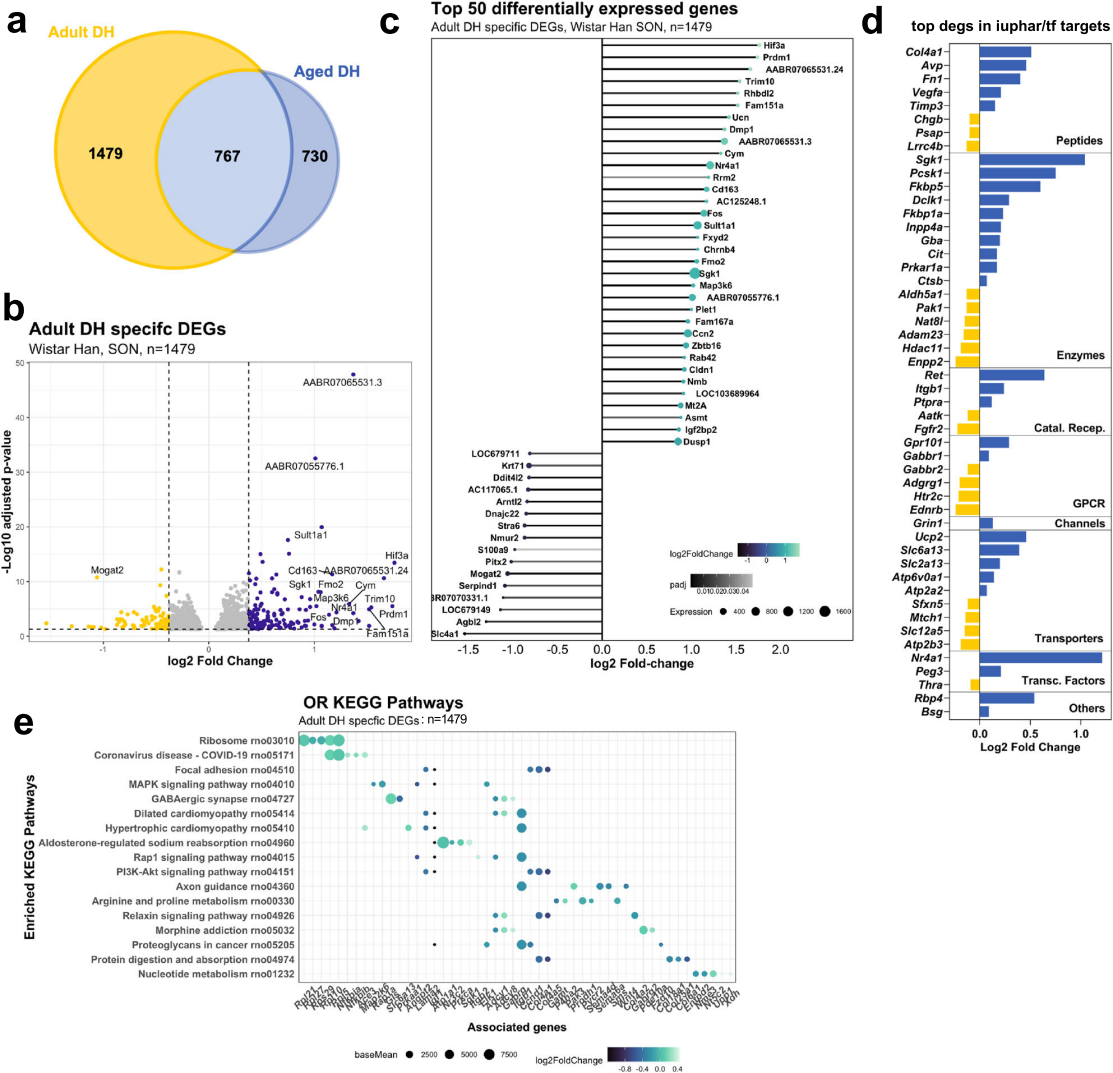
Comparison of adult DH DEGs with aged DH DEGs: unique adult DH DEGs. Venn comparison of adult DH and aged DH DEGs highlighting the unique adult DH DEGs (*n* = 1479) (**a**). Volcano plot showing the distribution of the unique adult DH DEGs (**b**). Lollipop graph highlighting the top 50 unique adult DH DEGs (**c**). Classification of the top 50 DEGS by LFC; Catal. Recep catalytic receptors, GPCR G protein-coupled receptor, Transc. Factors transcription factors (**d**). ORA KEGG pathway analysis of the unique adult DH DEGs (**e**).

We found that 1479 genes significantly change in expression as a consequence DH in adult rats but not in aged rats (Fig. 6a). These genes are displayed in a volcano plot using LFC of the adult DH dataset (Fig. 6b). The top 50 DEGs are presented organised according to LFC in a lollipop graph (Fig. 6c). DEGs were then catalogued according to their identity as transcription factors or their physiological or pharmacological classifications (Supplementary Data 13) (Fig. 6d). ORA KEGG analysis of the adult DH transcripts identified multiple enriched pathways such as ribosomes (rno03010; *P*_adj_ = 3.02E-08), MAPK signalling pathway (rno04010; *P*_adj_ = 6.5E-3), and aldosterone regulated sodium reabsorption (rno04960; *P*_adj_ = 1.7E-2) (Fig. 6e, Supplementary Data 14).

#### Unique aged DH DEGS

**Fig. 7:**
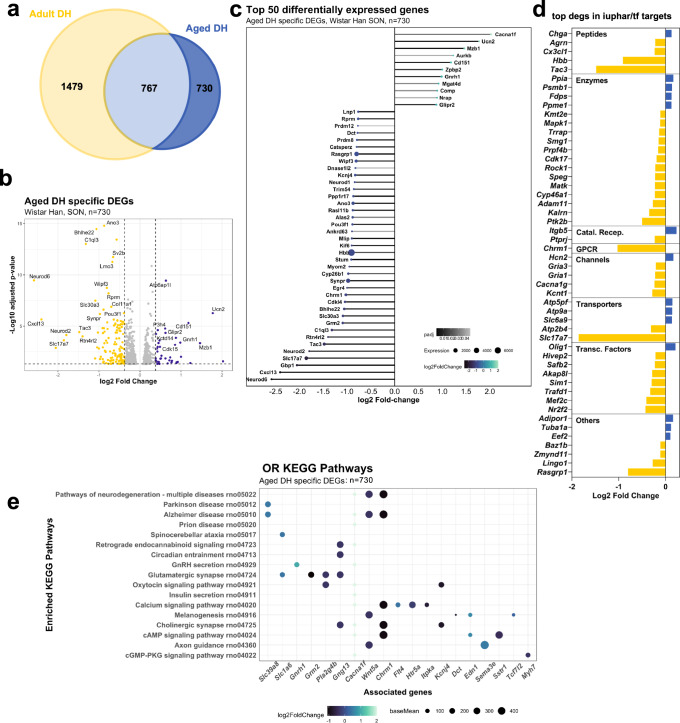
Comparison of adult DH DEGs with aged DH DEGs: unique aged DH DEGs. Venn comparison of adult DH and aged DH DEGs highlighting the unique aged DH DEGs (*n* = 730) (**a**). Volcano plot showing the distribution of the unique aged DH DEGs (**b**). Lollipop graph highlighting the top 50 unique aged DH DEGs (**c**). Classification of the top 50 DEGS by LFC; Catal. Recep catalytic receptors, GPCR G protein-coupled receptor, Transc. Factors transcription factors (**d**). ORA KEGG pathway analysis of the unique aged DH DEGs (**e**).

We found that 730 genes change in expression as a consequence DH in aged rats but not in adult rats (Fig. 7a). These genes are displayed in a volcano plot using LFC of the aged DH dataset (Fig. 7b). The top 50 DEGs are presented organised according to LFC in a lollipop graph (Fig. 7c). DEGs were then catalogued according to their identity as transcription factors or their physiological or pharmacological classifications (Supplementary Data 15) (Fig. 7d). ORA KEGG analysis revealed enrichment of pathways related to neurodegenerative diseases, including Parkinson’s (rno05012; *P*_adj_ = 1.9E-14), Alzheimer’s (rno5010; *P*_adj_ = 2.73E-14), prion (rno05020; *P*_adj_ = 9.37E-14), and spinocerebellar ataxia (rno05017; *P*_adj_ = 3.48E-6). Also, multiple signal pathways were affected, such as cAMP (rno04024; *P*_adj_ = 7.2E-3) and GMP-PKG (rno04022; *P*_adj_ = 7.9E-3) (Fig. 7e, Supplementary Data 16).

## Discussion

Good health is dependent upon the exact regulation of salt and water balance. These survival mechanisms are aggressively mobilised when osmotic stability is threatened. This is characterised by the activation of neuroendocrine mechanisms that control the excretion and consumption of water and salt in order to restore healthy bodily composition^8,9^. These mechanisms deteriorate with age, and dehydration is a common cause of morbidity and mortality in the elderly. Here we show that the process of natural ageing restructures the basal transcriptome of the SON of the rat, the core hypothalamic osmoregulatory control centre, and profoundly alters the response of the SON to dehydration.

We compared the transcriptomes of the SON in adult and aged euhydrated rats and found massive changes in gene expression associated with ageing. Functional ORA and WGCNA revealed enrichment for genes involved in extracellular matrix (ECM) organisation and cell adhesion. It is known that the SON has a complex and dynamic ECM that has been implicated in its physiological functioning. The SON undergoes dramatic activity-dependent structural plasticity as a consequence of sustained physiological stimulation^34–36^ characterised by increased direct neuronal membrane apposition, dendritic bundling and profound synaptic remodelling. SON glial cells actively participate in these plastic changes in association with MCNs. Crucial to these processes are cell adhesion molecules, belonging to the immunoglobulin superfamily, and extracellular matrix glycoproteins, which participate in neuronal-glial, neuronal-neuronal, and glial-glial recognition and guidance^65^. That the expression of genes encoding these classes of proteins changes with ageing suggests that intercellular interactions may be altered in the aged SON, perhaps related to the reported hypertrophy of glial perikarya and cell processes^66^.

One important consideration is how ageing affects the ensemble of channels that maintain resting membrane potential and produce action potentials (and thus synaptic release) in MCNs. For example, it is well established that SON neurons express Nav1.2 (encoded by the *Scn2a* gene) and Nav1.6 (encoded by the *Scb8a* gene) and the expression of these channels is upregulated with hyperosmolarity^67^. Nav1.7 (encoded by the *Scn9a* gene), which sets the gain on neurons via its action as a threshold channel, is also present in SON MCNs and is upregulated with osmolar challenge^68^. Examination of our ageing datasets suggests that the expression of these genes is not affected by ageing. Rather, any changes in the electrical activity of the SON with ageing would likely be mediated by purinergic receptors (*P2rx4*, *P2rx5*, both upregulated by ageing), gap junction proteins (*Gja1*, *Gjb2*, *Gjb6*, *Gjc1*, *Gjc3*, all downregulated by ageing), a hyperpolarization-activated cyclic nucleotide-gated potassium channel (*Hcn3*, upregulated by ageing), or transient receptor potential cation channels (*Trpc4*, *Trpm2*, both upregulated by ageing) (Supplementary Table 3). Although microarray analysis revealed upregulation of *Trpc4* in the whole SON following 72 h of dehydration^49^, downregulation of *Trpc4* expression was found specifically in AVP SON MCNs in young adult animals subjected to 48 h of water deprivation^69^.

We have previously described how the adult SON transcriptome responds to denydration^53^. Here we report how the transcriptome of the aged SON responds to dehydration. Interestingly, ORA analysis revealed that gene sets were enriched in terms related to neurodegenerative processes and diseases. This is perhaps paradoxical, given that the SON has been described as being resistant to deterioration consequential to Alzheimer’s disease (AD)^23–33^, but might suggest that the process of dehydration itself provokes degenerative consequences.

Dysfunction water homoeostasis in ageing is associated with the inappropriate release of the antidiuretic hormone AVP^70^. In both humans and rodents, AVP MCNs undergo morphological changes as they age that are indicative of hyperactivity, including increased size of perikarya, nucleoli and Golgi apparatus^71^. These morphological changes are similar to those seen in MCNs subject to dehydration^72^. Increased transcription and protein synthesis is needed to make more neurosecretory products, and this results in cellular hypertrophy. It has been suggested that MCN hyperactivity may lead to electrolyte disorders in the elderly^70^. Transcriptional changes have also been reported in the aged SON^20^, some of which are indicative of neuronal activation. For example, the expression of the neuronal plasticity marker gene *Fos* is increased in the aged SON^20,73^. We examined the relationship between ageing and physiological activation at the whole transcriptome level by comparing ageing DEGs with both adult and ageing DH DEGs. We found that 384 genes that change in expression as a consequence of DH in adult rats are also altered by ageing. ORA revealed that these genes are involved in lysosomal function, ECM-receptor interaction, protein processing in the endoplasmic reticulum, and longevity. Comparison of the ageing DEGS with the aged DH DEGs revealed 212 common genes with enrichment for pathways related to lysosomal function, endoplasmic reticulum protein processing and stress, and the recruitment of aminoacyl tRNAs. Thus, it would appear that ageing in the SON involves the regulation of gene networks that are common to both adult and aged DH. These networks are involved in the synthesis and delivery of considerable quantities of secretory protein products, consistent with the previously reported hyperactivity of aged SON neurones^70,71,73^.

Comparison of the adult SON DH DEGS with those of the aged SON revealed that the transcriptomic response to DH is very different. It is noteworthy that a large number of genes that are significantly altered in expression as a consequence of dehydration in the adult animal do not respond to dehydration in the aged rat. These genes are enriched for functionalities related to translation (ribosomes), the MAPK signalling pathway, and aldosterone actions that are presumably lost in the aged animal. A smaller number of genes are altered in expression as a consequence of DH only in the aged SON. ORA again revealed enrichment for terms involved in neurodegeneration that are not evident in that adult animal as well as terms associated with signalling pathways involving cAMP and GMP-PKG.

The capabilities of the AVP system to respond to osmotic stress decrease with age^74–76^. In the aged animal, the capacity of the AVP system to respond to dehydration is attenuated^77^. These deficits may be associated with dysfunction in mechanisms controlling transcription, mRNA stability or translation^78^. Indeed, we have previously shown that the steady-state response to dehydration of a number of selected gene transcripts (*Fos, Creb3l1, Giot1, Caprin2, Rasd1, Slc12a1*) is attenuated in aged animals^20^. This appears to be a transcriptome level effect, with many of the common genes regulated by dehydration showing a blunted response in aged animals compared to adults (Fig. 5). This generalised attenuation of the transcriptomic response to dehydration is likely to greatly affect SON function and overall osmoregulatory effectiveness under these challenging physiological and pathophysiological conditions.

Although we have previously employed microarrays to catalogue and compare differences in the transcriptomic response to dehydration of adult male and female SON^39^, in this ageing study, we only examined male rats. Given that age-related sex differences in osmoregulation in the rat have been described^78,79^, this is a shortcoming of this study. In this context, it is important to note that in young humans, both sexes have the same number of AVP neurones^80^, but these neurones are larger in males compared to females^81^. This perhaps contributes to the elevated levels of circulating AVP in young males compared to females. With age, the size of AVP neurones increases in females but not in males, and this results in increased circulating AVP levels in females with age^82,83^.

Rats were harvested at a single time point in the daytime (10 am–12 noon). In young humans, AVP is secreted with a diurnal rhythm with a strong peak during the night^84,85^. This nocturnal peak diminishes during the course of healthy ageing^84–87^. In the rat, a similar diurnal rhythm has been observed^88,89^. Plasma AVP levels peak at dusk and reach a nadir at dawn, with pituitary content showing an inverse pattern^88,89^. The effect of ageing on this rhythm in rats has not been studied and was not addressed in our study.

Genome sequencing projects have revealed that approximately 22,000 protein-coding genes are needed to make a mammal. However, a tiny proportion of these genes have been studied in-depth. Remarkably, 5% of genes dominate 70% of neuroscience publications^90^. The majority of genes belong to the ignorome^91^, the extent of which reduces the biological value of GO analyses. Given that 58% of annotations are represented by only 16% of human genes^92^. GO and pathway analysis is intrinsically limited because most genes are not annotated with any functional information; 58% of annotations are represented by only 16% of human genes^92^. In this context, it is amusing to note that the Coronavirus disease-COVID-19 pathway (rno05171) is represented in one of our OR-KEGG analyses (Fig. 6e). This bias simply reflects the extent of recent research in this area^93^.

Whilst transcriptomics can provide considerable global information regarding steady-state mRNA levels, it must be conceded that it is the proteome that is the engine of the cell. Physiological extrapolations from transcriptome data must therefore be interpreted with caution.

Finally, we acknowledge that this bulk RNAseq analysis, whilst focusing on the SON, does not address the cellular heterogeneity of this discrete hypothalamic nucleus. We note that one study has carried out a single nuclei RNAseq analysis of the ageing female mouse hypothalamus^94^. However, limited data on the SON precluded the deconvolution of our transcriptomes. Remining of these data revealed only seven genes as being significantly changed as a consequence of ageing in the small number (only ~100 in each age group) of identified female mouse MCNs. None of these genes overlapped with our ageing SON dataset, possibly due to sex or species differences or technical issues and limitations related to different methodologies. For example, our bulk SON transcriptome analyses revealed the over-expression of *Oxt* in response to ageing. On the other hand, Hajdarovic et al.^94^ found a reduction in *Oxt*-derived RNA in the aged hypothalamus when treating their single-nucleus RNAseq as bulk. This apparent discrepancy might be related to many factors, such as differences in RNA processing, changes in nucleus-to-cytoplasm transportation and sex dimorphism of hypothalamic transcriptomic responses to ageing, amongst others.

It is now well recognised that disorders of body salt and water composition become more commonplace as we age. Changes in the vasopressin system with ageing, as described here at the transcriptome level, are thus potentially of great importance clinically and may be of potential prognostic and therapeutic value. We reveal that healthy ageing results in numerous robust changes in the transcriptomic landscape of the SON, with extracellular matrix components being particularly prominent. Overall, the response to dehydration in aged animals appears to be blunted, but intriguingly DEGs associated with neurodegenerative processes become apparent, raising the possibility that long-life episodes of dehydration may impact the development of neurodegenerative diseases such as Alzheimer’s or Parkinson’s.

## Methods

### Animals

All experiments were performed under the auspices UK Home Office Project Licence held under and in strict accordance with the provisions of the UK Animals (Scientific Procedures) Act (1986); they were also approved by the University of Bristol Animal Welfare and Ethical Review Board. We choose to use male Wistar Han rats from the international standardisation programme (IGS) in our study (Charles River). This carefully managed breeding programme minimises the impact of genetic drift so that colonies bred in different locations around the world are not significantly divergent from each other, giving a level of continuity in studies performed in laboratories worldwide. A total of 24 rats aged (18 months old) were purchased for this study. Rats were housed in groups of three at a constant temperature (22 °C) and a relative humidity of 50–60% (v/v) under a 14:10-h light/dark cycle (lights on at 0500) with food and water ad libitum for 2 weeks. Cages contained sawdust, bedding material, and cardboard tubing for enrichment. Rat cages were randomly assigned to two groups of 12 animals: EH (free access to drinking water) and DH (removal of drinking water for 72 h). All rats were humanely killed by striking the cranium (stunning) and then immediately decapitated with a small animal guillotine (Harvard Apparatus). Brains were rapidly removed from the cranium and placed into a chilled rat brain matrix for separation of the forebrain from the hindbrain. The forebrain was placed cut edge down onto aluminium foil resting on pellets of dry ice and covered with powdered dry ice (within 3 min of stunning). Animal experiments were performed between 10 am and 12 pm. Studies on young (adult) and aged rats were performed in parallel under the exact same experimental conditions to ensure direct comparability of these datasets and to obviate the need for any batch correction. All sampling was performed by the same team of investigators. All adult and aged rat SON isolations, by punching, were performed by the same researcher at the same time. RNA isolations from adult and aged rat SONs were performed by the same researcher at the same time. Adult and aged SON RNA samples were processed by Source Bioscience at the same time for RNAseq.

### Plasma AVP measures

Trunk blood plasma was collected in 1 ml aliquots, snap-frozen in liquid nitrogen and stored at −80 °C. Plasma was extracted by vortexing for 1 min with two sample volumes of ice-cold acetone. Precipitates were removed by centrifugation at 2500 × *g*, 4 °C, for 25 min, and supernatants were transferred to a fresh tube, then mixed by vortexing for 1 min with 2 ml of cold petroleum ether. Tubes were allowed to rest at room temperature for 1 min before discarding the upper phase. The lower phase was lyophilised in a freeze dryer (Benchtop Pro, Biopharma). A specific radioimmunoassay was used to quantify plasma AVP concentrations^95^.

### Plasma osmolality measures

Trunk blood was collected in heparin-coated tubes, and the plasma supernatant was derived by centrifugation at 1600 × *g* for 15 min at 4 °C. A Roebling micro-osmometer was used to measure plasma osmolality using the freezing point depression method (Camlab).

### SON RNA extraction

Using the optic chiasm as a reference, a 1-mm sample corer (Fine Scientific Tools) was used to collect bilateral punches of the SON from hypothalamic coronal slices (Fine Scientific Tools). SON punches were frozen on dry ice, then re-suspended by continuous vortexing for 1 min in 400 µl of QIAzol lysis reagent (Qiagen). Following a 10-min incubation at room temperature, debris was removed by centrifugation at 12,000 × *g* for 3 min. The supernatant was carefully removed and then mixed with an equal volume of absolute ethanol. Total RNA was extracted with the Direct-zol RNA MiniPrep extraction kit (Zymo Research, Irvine, CA, USA) and then applied to a Zymo-Spin IIC column. RNA was eluted in a volume of 25 µl.

### Reverse transcription (RT) quantitative polymerase chain reaction (RT-qPCR)

For cDNA synthesis, 40 ng of total RNA was reverse transcribed using the QuantiTect reverse transcription (RT) kit (Qiagen). Primers for rat genes used in this study can be found in Supplementary Table 2 (Eurofins MWG Operon). The optimisation and validation of primers were performed using standard Applied Biosystems protocols. The cDNA from the RT reaction was diluted 1:4 with H_2_O and used as a template for subsequent polymerase chain reactions (PCRs), which were carried out in duplicate using SYBR green (Roche) on an Applied Biosystems StepOnePlus Real-Time PCR system. For relative quantification of gene expression, the 2^−^^ΔΔCT^ method was used^96^. The internal control housekeeping gene used for these analyses was the housekeeping gene *Gapdh*.

### RNA sequencing (RNAseq)

Rigorous quality control checks were applied to assess the purity and integrity of RNA (Agilent BioAnalyzer; RNA TapeStation) RIN values ranged between 8.4 and 8.8 (see Supplementary Table 3). Illumina TruSeq Stranded mRNA kits were used to generate Poly(A) enriched bulk RNA-sequencing libraries. These libraries were loaded onto lanes of an Illumina NextSeq flowcell and sequenced using 75 bp paired-end (PE) runs (Source Bioscience). Each individual sample generated >35 million PE reads. Data were analysed as described in detail^53^. The analysis was sufficiently powered (*n* = 5 per group) to reduce the false discovery rate and to enable systems-level analysis^97^. Differentially expressed genes (DEGs) with *P*-adjusted (*P*_adj_) values of <0.05 are considered significant.

### RNAseq data mining

RNAseq data mining pipelines have been described in detail^53^. Briefly, ENSEMBL was used for genome annotations using biomaRt (v2.44.4), org.Rn.eg.db (v3.11.4), AnnotationDbi (v1.50.3), Bioconductor (v3.11.4) packages in R. Scaled Venn diagrams were generated using VennDiagram (version 1.6.20) package in R. Enrichment analysis was performed using ClusterProfiler (v3.10.1) and ReactomePA (v1.32.0) packages in R. WGCNA (v4.0.3) package was used for the network correlation analysis. Benjamini–Hochberg correction (*P*_adj_ < 0.05) was used for multiple comparison corrections in enrichment analysis. Gene expression data were visualised using custom scripts written in R using the “ggplot2” (v3.3.5) package.

### Reporting summary

Further information on research design is available in the Nature Research Reporting Summary linked to this article.

## Supplementary information

Reporting Summary

Supplementary Information

Supplementary Data set 1

Supplementary Data set 2

Supplementary Data set 3

Supplementary Data set 4

Supplementary Data set 5

Supplementary Data set 6

Supplementary Data set 7

Supplementary Data set 8

Supplementary Data set 9

Supplementary Data set 10

Supplementary Data set 11

Supplementary Data set 12

Supplementary Data set 13

Supplementary Data set 14

Supplementary Data set 15

Supplementary Data set 16

## Data Availability

Transcriptomic datasets are publically accessible (https://www.ncbi.nlm.nih.gov/geo/query/acc.cgi?acc=GSE214353).

## Code availability

RNAseq data mining pipelines have been described in detail^53^. Please contact the corresponding authors for access to custom codes.
