# Supplementary material for: Ageing restructures the transcriptome of the hypothalamic supraoptic nucleus and alters the response to dehydration
Source: NPJ Aging. 2023 Jun 1;9(1):12. doi: 10.1038/s41514-023-00108-2 (PMC10234251; doi:10.1038/s41514-023-00108-2)
Supplement: Supplementary file 2 — Supplementary Information [file 41514_2023_108_MOESM2_ESM.pdf]

Supplementary Figure 1

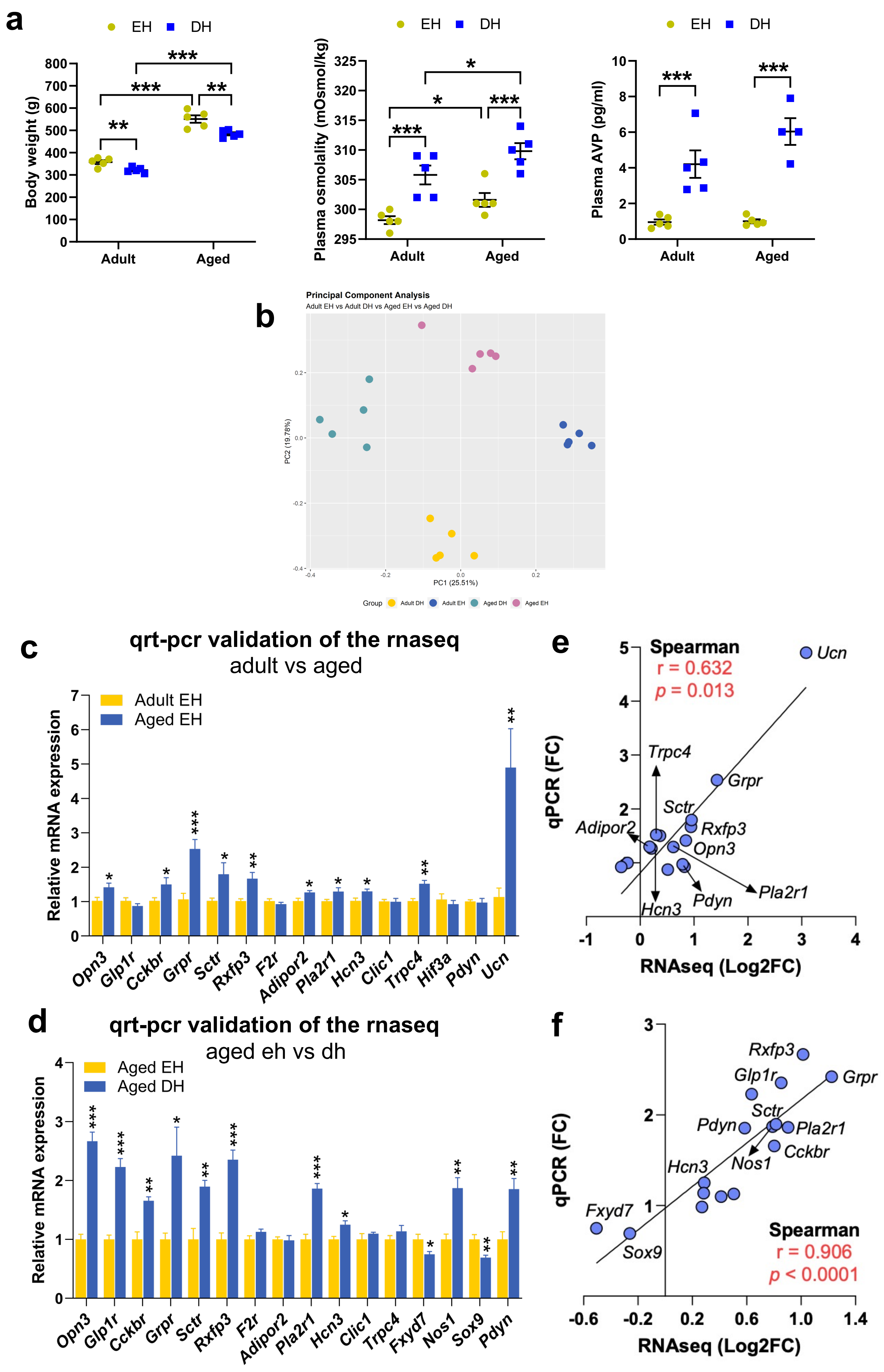

g

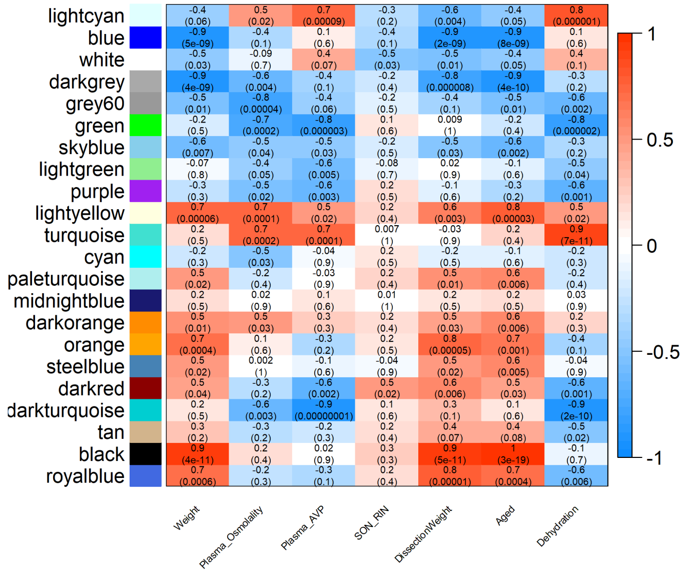

n

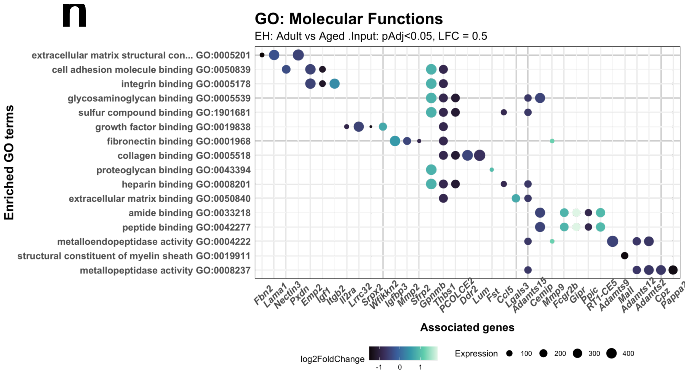

i

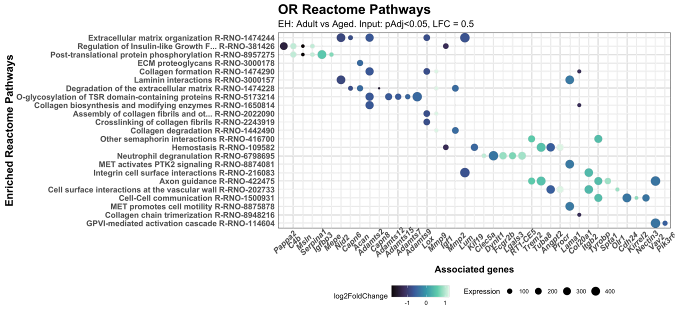

Supplementary Figure 1. Body weight was documented before and after DH and plasma osmolality and plasma AVP were measured (A). Analysis by two-way ANOVA with Tukey's post hoc test. A Grubbs' test identified a significant outlier for aged DH AVP with an Alpha = 0.05 which was removed (22.9 pg/ml). PCA plot illustrating the distinct differences between all four groups in this study (B). RT-qPCR validation of DEGs in ageing EH SON (comparing adult EH and aged EH) (C) and DH in SON of aged animals (comparing aged EH and aged DH) (D). Analysis by unpaired t-test. Spearman correlations comparing the RT-qPCR and RNAseq expression data from the ageing EH SON (comparing adult EH and aged EH) (E) and DH in SON of aged animals (comparing aged EH and aged DH) (F). WGCNA module-trait association matrix (G). Each row corresponds to a module labeled with a colour, and each column corresponds to a physiological trait. Dark red shows strong positive correlation whilst dark blue shows strong negative correlation. Dot plot showing GO ORA analysis of the MF terms (H) and Reactome pathway analysis (I) and their associated DEGs derived from the ageing DEGs. Values are means +/- SEM of n=5. *$p<0.05$, **$p<0.01$, ***$P<0.001$.

Supplementary Table 1 - Definitions of gene symbols

Ensembl_gene_ID	entrezgene_ID	Gene_name	description		
ENSRNOG00000031216	NA	AABR07055776.1	NA		
ENSRNOG00000041744	NA	AABR07065531.1	NA		
ENSRNOG00000004731	311287	Ano3	anoctamin 3		
ENSRNOG00000017801	79255	Atf4	activating transcription factor 4		
ENSRNOG00000040201	361875	Atp6ap1l	ATPase H+ transporting accessory protein 1 like		
ENSRNOG00000021229	24221	Avp	arginine vasopressin		
ENSRNOG00000012906	246755	Bcas1	breast carcinoma amplified sequence 1		
ENSRNOG00000047300	25245	Bdkrb2	bradykinin receptor B2		
ENSRNOG00000055962	25181	Bgn	biglycan		
ENSRNOG00000021745	365748	Bhlhe22	basic helix-loop-helix family, member e22		
ENSRNOG00000034174	499925	Bpifb4	BPI fold containing family B, member 4		
ENSRNOG00000017459	680404	C1ql3	complement C1q like 3		
ENSRNOG00000060020	300220	C1ql4	complement C1q like 4		
ENSRNOG00000046834	24232	C3	complement C3		
ENSRNOG00000058938	79011	Camkv	CaM kinase-like vesicle-associated		
ENSRNOG00000047319	686779	Caprin2	caprin family member 2		
ENSRNOG00000019215	64315	Cd151	CD151 molecule (Raph blood group)		
ENSRNOG00000040266	503009	Cdkl4	cyclin-dependent kinase-like 4		
ENSRNOG00000059500	246060	Cdkn1c	cyclin-dependent kinase inhibitor 1C		
ENSRNOG00000056996	301521	Cfap65	cilia and flagella associated protein 65		
ENSRNOG00000018385	25229	Chrm1	cholinergic receptor, muscarinic 1		
ENSRNOG00000026870	304081	Clic6	chloride intracellular channel 6		
ENSRNOG00000003897	29393	Col1a1	collagen type I alpha 1 chain		
ENSRNOG00000018951	363457	Col4a5	collagen type IV alpha 5 chain		
ENSRNOG00000005413	362165	Creb3l1	cAMP responsive element binding protein 3-like 1		
ENSRNOG00000050697	252929	Ctsz	cathepsin Z		
ENSRNOG00000024899	498335	Cxcl13	C-X-C motif chemokine ligand 13		
ENSRNOG00000008671	290484	Dct	dopachrome tautomerase		
ENSRNOG00000008015	314322	Fos	Proto-oncogene c-Fos		
ENSRNOG00000020552	25445	Fosl1	FOS like 1, AP-1 transcription factor subunit		
ENSRNOG00000051563	171090	Giot1	gonadotropin inducible ovarian transcription factor 1		
ENSRNOG00000015860	25024	Gipr	gastric inhibitory polypeptide receptor		
ENSRNOG00000014838	679819	Glipr2	GLI pathogenesis-related 2		
ENSRNOG00000056457	60666	Gpd1	glycerol-3-phosphate dehydrogenase 1		
ENSRNOG00000054204	29627	Gria2	glutamate ionotropic receptor AMPA type subunit 2		
ENSRNOG00000001251	117130	Grifin	galectin-related inter-fiber protein		
ENSRNOG00000017198	64345	Hif3a	Hypoxia-inducible factor 3 alpha		
ENSRNOG00000018294	25617	Hspa5	heat shock protein family A (Hsp70) member 5		
ENSRNOG00000007066	25689	Htr5a	5-hydroxytryptamine receptor 5A		
ENSRNOG00000004517	24482	Igf1	insulin-like growth factor 1		
ENSRNOG00000020369	24483	Igf2	insulin-like growth factor 2		
ENSRNOG00000025946	303824	Igf2bp2	insulin-like growth factor 2 mRNA binding protein 2		
ENSRNOG00000016957	25662	Igfbp2	insulin-like growth factor binding protein 2		
ENSRNOG00000001224	309684	Itgb2	integrin subunit beta 2		
ENSRNOG00000047768	25473	Lamb2	laminin subunit beta 2		
ENSRNOG00000047450	497798	Lmo3	LIM domain only 3		
ENSRNOG00000049918	367455	Lrg1	leucine-rich alpha-2-glycoprotein 1		
ENSRNOG00000015599	362211	Mall	mal, T-cell differentiation protein-like		
ENSRNOG00000006209	362892	Mbd6	methyl-CpG binding domain protein 6		
ENSRNOG00000027228	681211	Mogat2	Monoacylglycerol O-Acyltransferase 2		
ENSRNOG00000026055	500137	Neurod6	neuronal differentiation 6		
ENSRNOG00000000341	302248	Nid2	nidogen 2		
ENSRNOG00000021225	25504	Oxt	Oxytocin		
ENSRNOG00000042860	680415	Pappa2	pappalysin 2		
ENSRNOG00000022162	311876	Pbx3	PBX homeobox 3		
ENSRNOG00000019265	116808	Pcdh12	protocadherin 12		
ENSRNOG00000026036	29190	Pdyn	prodynorphin		
ENSRNOG00000037931	50692	Plaur	plasminogen activator, urokinase receptor		
ENSRNOG00000019330	362248	Procr	protein C receptor		
ENSRNOG00000052880	24688	Prph	peripherin		
ENSRNOG00000006320	59103	Ptges	prostaglandin E synthase		
ENSRNOG00000003253	64192	Qdpr	quinoid dihydropteridine reductase		
ENSRNOG00000039754	501854	Rab7b	Rab7b, member RAS oncogene family		
ENSRNOG00000003348	64455	Rasd1	Ras related dexamethasone induced 1		
ENSRNOG00000005367	25065	Slc12a1	solute carrier family 12 member 1		
ENSRNOG00000057256	316559	Slc19a3	solute carrier family 19 member 3		
ENSRNOG00000006204	366568	Slc30a3	solute carrier family 30 member 3		
ENSRNOG00000015159	24784	Slc9a3	solute carrier family 9 member A3		
ENSRNOG00000019342	83783	Sult1a1	Sulfotransferase Family, Cytosolic, 1A, Phenol-Preferring		
ENSRNOG00000011160	117556	Sv2b	synaptic vesicle glycoprotein 2b		
ENSRNOG00000058645	116640	Tnc	tenascin C		
ENSRNOG00000050792	84397	Tnfaip6	TNF alpha induced protein 6		
ENSRNOG00000006090	29151	Ucn	urocortin		
ENSRNOG00000020655	170896	Ucn2	urocortin 2		
ENSRNOG00000001416	29461	Vgf	VGF nerve growth factor inducible		

Supplementary Table 2: qRT-PCR Primers used in the validation of the selected genes

Ensembl ID	Gene	Forward 5'-3'	Reverse 5'-3'
ENSRNOG00000003778	Opn3	GTGAGCTCCACCCGGTTC	TGTGTCGCAGCTGAGGTAAG
ENSRNOG00000001152	Glp1r	GTTCCGCTGCTGTTCGTTAT	GCAAGCGTATGATGAGCCAA
ENSRNOG00000017679	Cckbr	AACAAACCTGGTCCGTGCTA	CCATAGGCCACCGCAATAAC
ENSRNOG00000004124	Grpr	TGGCTGCAAACTGATCCCCTT	CATTGGCCTGACGATGGCTTT
ENSRNOG00000049766	Sctr	TGCTCCTCGGATGGGGTTCT	GACGGAAGCATTGGCGTTGA
ENSRNOG00000023126	Rxfp3	GACAAGCTCCTGGGTTGGGA	AGATGAAGCGCACGAGCAAC
ENSRNOG00000048043	F2r	TGGATTCTGCGCTTTGACGC	CCAGGGCTAGCATCCACCAA
ENSRNOG00000007990	Adipor2	GCTGGGCATTGCAGCCATTAT	TCAGACCCAAGCCCACGAAC
ENSRNOG00000008129	Pla2r1	CCCCATAAGTGGAGCGCCTA	CGTGGTGCCACTGACCTTTG
ENSRNOG00000020444	Hcn3	CACTCGCCTCACTGACGGAT	GTGGTCCACGCTGAGTGAGT
ENSRNOG00000029682	Clic1	GAATTCGCCTCCACCTGTCCT	ATTTGAGAGCCCTGGCCACTT
ENSRNOG00000011133	Trpc4	CAGATGTGGGATGGCGGACT	GTCCCATGATTCCCGTGGGT
ENSRNOG00000017198	Hif3a	GGGAGTGATCCACGACTGAA	CTTCCGAGTTCTAGGCGTCA
ENSRNOG00000026036	Pdyn	TGGATCGGCCATCCTATCAC	GCAGATCTCAAAGCCTGTGG
ENSRNOG00000006090	Ucn	GCACTCCTCTTGCTGTTAGC	AAGGTGAGGTCGATGGACAA
ENSRNOG00000021067	Fxyd7	CCAGGTCTGAGAGCCCAACA	GATCCTACACACCGCCACCT
ENSRNOG00000001130	Nos1	GCTGAAGGGCCTCTATGCTA	CTCTCGGTAGCCACAGTACC
ENSRNOG00000002607	Sox9	TCTGAGCTCATGCAAACACG	TCCAAACAGGCAGGGAGATT

Supplementary Table 3. RNA yield and integrity used for RNAseq

Animal ID	Condition	RIN scores	Total RNA yield (ng/μl)
A1	Aged EH	8.8	7.1
A2	Aged EH	8.4	7.6
A4	Aged EH	8.8	7.9
A5	Aged EH	8.5	9.5
A6	Aged EH	8.5	10.9
B1	Aged DH	8.7	11
B2	Aged DH	8.7	8
B3	Aged DH	8.7	9.6
B5	Aged DH	8.4	12.4
B6	Aged DH	8.7	14.1
C1	Adult EH	8.6	8.8
C2	Adult EH	8.6	10.1
C4	Adult EH	8.6	8.7
C5	Adult EH	8.6	8.3
C6	Adult EH	8.6	8.6
D1	Adult DH	8.6	11.1
D2	Adult DH	8.6	10.8
D3	Adult DH	8.5	12
D4	Adult DH	8.5	9.4
D5	Adult DH	8.4	10.4

Supplementary Data Legends

Supplementary Data 1 Ageing and aged DH SON RNAseq datasets. Full datasets are presented alongside significant DEGs.

Supplementary Data 2 Classification of ageing DEGs.

Supplementary Data 3 GO (MF) terms and genes enriched in the ageing DEGs.

Supplementary Data 4 Reactome terms and genes enriched in the ageing DEGs.

Supplementary Data 5 KEGG terms and genes enriched in the ageing DEGs.

Supplementary Data 6 Classification of aged DH DEGs.

Supplementary Data 7 KEGG terms and genes enriched in the aged DH DEGs.

Supplementary Data 8 Classification of common ageing and adult DH DEGs.

Supplementary Data 9 KEGG terms and genes enriched in the common ageing and adult DH DEGs.

Supplementary Data 10 Classification of common ageing and aged DH DEGs.

Supplementary Data 11 Classification of common adult DH and aged DH DEGs.

Supplementary Data 12 KEGG terms and genes enriched in the common adult DH and aged DH DEGs.

Supplementary Data 13 Classification of unique adult DH DEGs.

Supplementary Data 14 KEGG terms and genes enriched in the unique adult DH DEGs.

Supplementary Data 15 Classification of unique aged DH DEGs.

Supplementary Data 16 KEGG terms and genes enriched in the unique aged DH DEGs.
