# Supplementary figures and images for: Ageing restructures the transcriptome of the hypothalamic supraoptic nucleus and alters the response to dehydration
Source: NPJ Aging. 2023 Jun 1;9(1):12. doi: 10.1038/s41514-023-00108-2 (PMC10234251; doi:10.1038/s41514-023-00108-2)

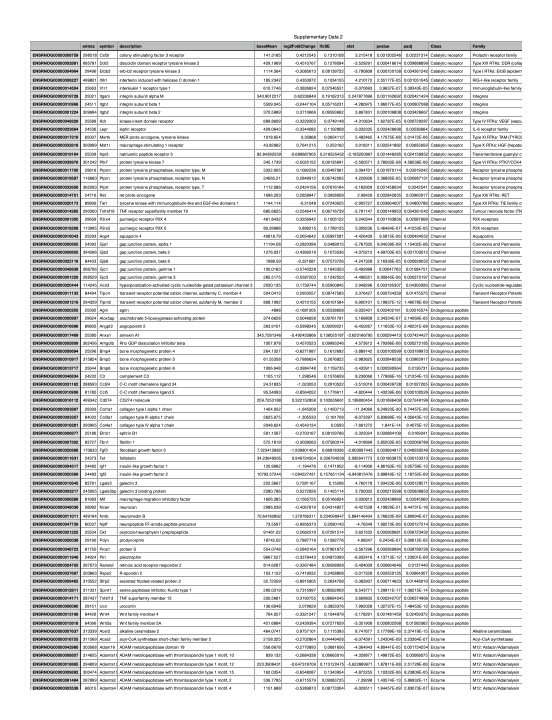

Supplement: Supplementary file 4 — Supplementary Data set 2 [file 41514_2023_108_MOESM4_ESM.zip › preview.jpg]

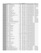

Supplement: Supplementary file 4 — Supplementary Data set 2 [file 41514_2023_108_MOESM4_ESM.zip › preview-micro.jpg]

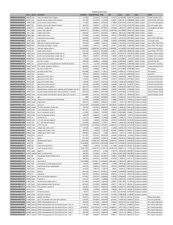

Supplement: Supplementary file 4 — Supplementary Data set 2 [file 41514_2023_108_MOESM4_ESM.zip › preview-web.jpg]

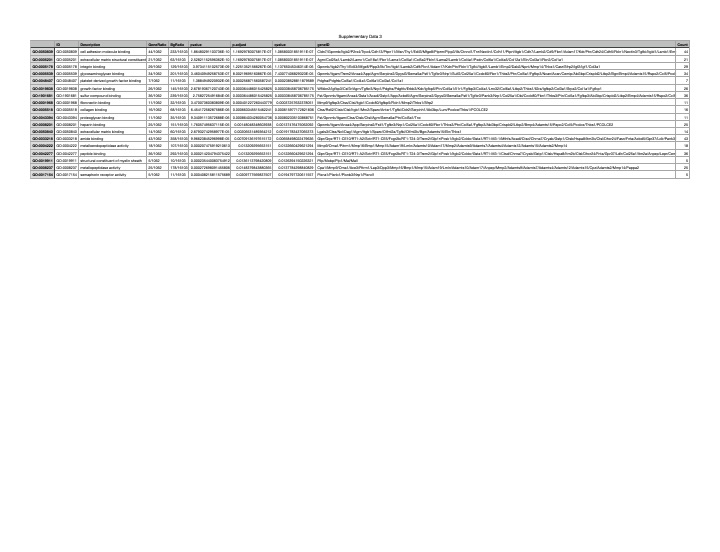

Supplement: Supplementary file 5 — Supplementary Data set 3 [file 41514_2023_108_MOESM5_ESM.zip › preview.jpg]

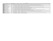

Supplement: Supplementary file 5 — Supplementary Data set 3 [file 41514_2023_108_MOESM5_ESM.zip › preview-micro.jpg]

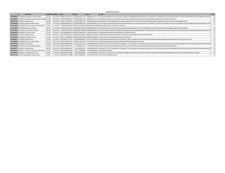

Supplement: Supplementary file 5 — Supplementary Data set 3 [file 41514_2023_108_MOESM5_ESM.zip › preview-web.jpg]

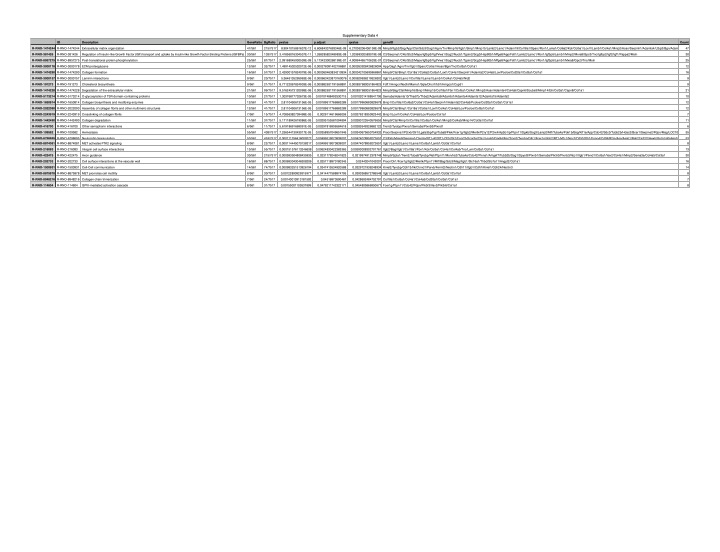

Supplement: Supplementary file 6 — Supplementary Data set 4 [file 41514_2023_108_MOESM6_ESM.zip › preview.jpg]

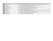

Supplement: Supplementary file 6 — Supplementary Data set 4 [file 41514_2023_108_MOESM6_ESM.zip › preview-micro.jpg]

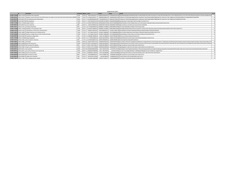

Supplement: Supplementary file 6 — Supplementary Data set 4 [file 41514_2023_108_MOESM6_ESM.zip › preview-web.jpg]

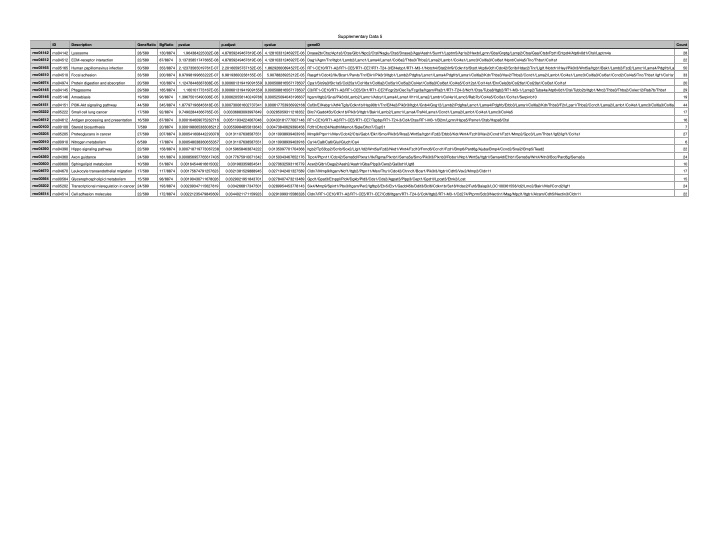

Supplement: Supplementary file 7 — Supplementary Data set 5 [file 41514_2023_108_MOESM7_ESM.zip › preview.jpg]

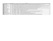

Supplement: Supplementary file 7 — Supplementary Data set 5 [file 41514_2023_108_MOESM7_ESM.zip › preview-micro.jpg]

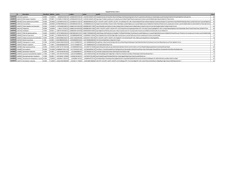

Supplement: Supplementary file 7 — Supplementary Data set 5 [file 41514_2023_108_MOESM7_ESM.zip › preview-web.jpg]

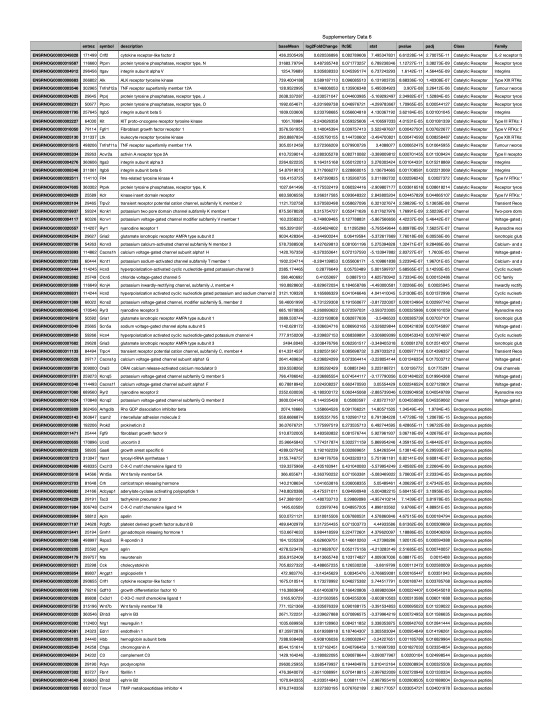

Supplement: Supplementary file 8 — Supplementary Data set 6 [file 41514_2023_108_MOESM8_ESM.zip › preview.jpg]

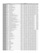

Supplement: Supplementary file 8 — Supplementary Data set 6 [file 41514_2023_108_MOESM8_ESM.zip › preview-micro.jpg]

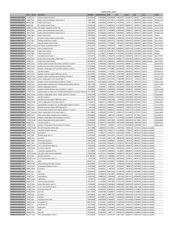

Supplement: Supplementary file 8 — Supplementary Data set 6 [file 41514_2023_108_MOESM8_ESM.zip › preview-web.jpg]

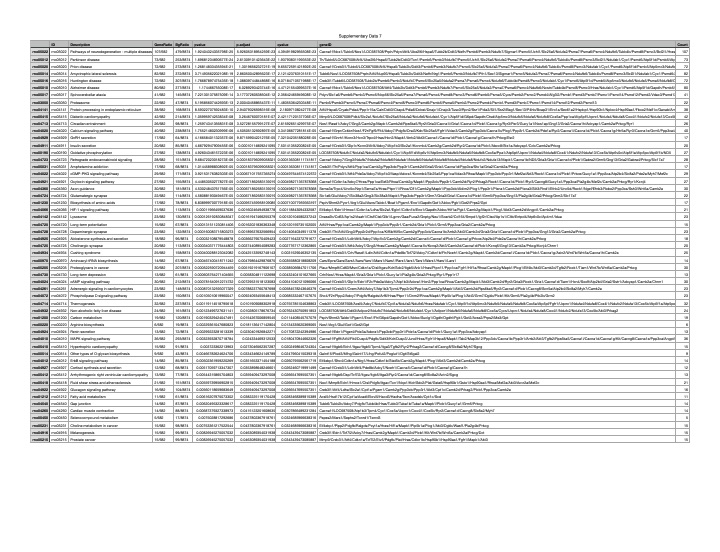

Supplement: Supplementary file 9 — Supplementary Data set 7 [file 41514_2023_108_MOESM9_ESM.zip › preview.jpg]

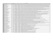

Supplement: Supplementary file 9 — Supplementary Data set 7 [file 41514_2023_108_MOESM9_ESM.zip › preview-micro.jpg]

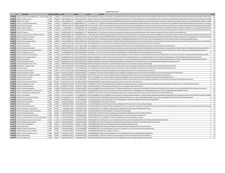

Supplement: Supplementary file 9 — Supplementary Data set 7 [file 41514_2023_108_MOESM9_ESM.zip › preview-web.jpg]

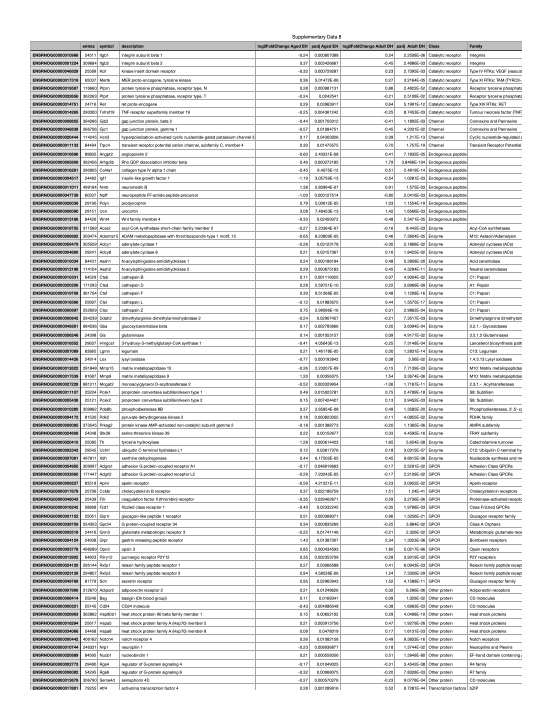

Supplement: Supplementary file 10 — Supplementary Data set 8 [file 41514_2023_108_MOESM10_ESM.zip › preview.jpg]

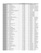

Supplement: Supplementary file 10 — Supplementary Data set 8 [file 41514_2023_108_MOESM10_ESM.zip › preview-micro.jpg]

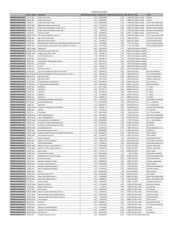

Supplement: Supplementary file 10 — Supplementary Data set 8 [file 41514_2023_108_MOESM10_ESM.zip › preview-web.jpg]

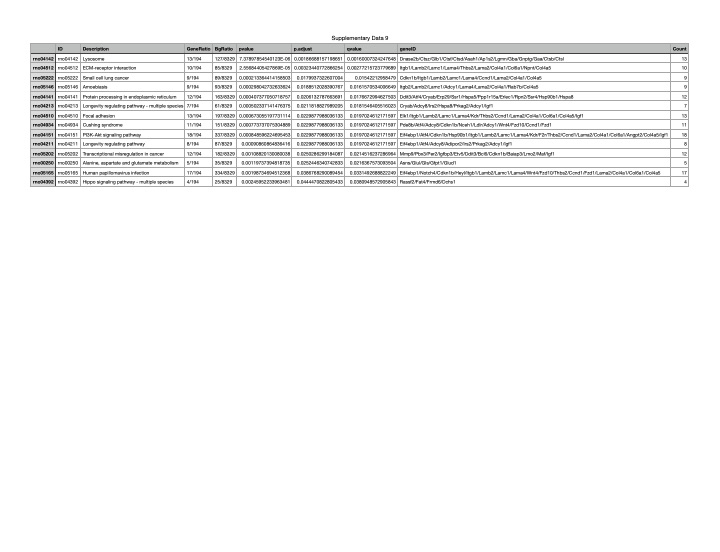

Supplement: Supplementary file 11 — Supplementary Data set 9 [file 41514_2023_108_MOESM11_ESM.zip › preview.jpg]

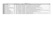

Supplement: Supplementary file 11 — Supplementary Data set 9 [file 41514_2023_108_MOESM11_ESM.zip › preview-micro.jpg]

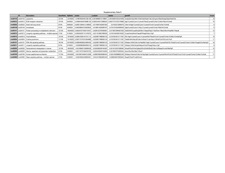

Supplement: Supplementary file 11 — Supplementary Data set 9 [file 41514_2023_108_MOESM11_ESM.zip › preview-web.jpg]

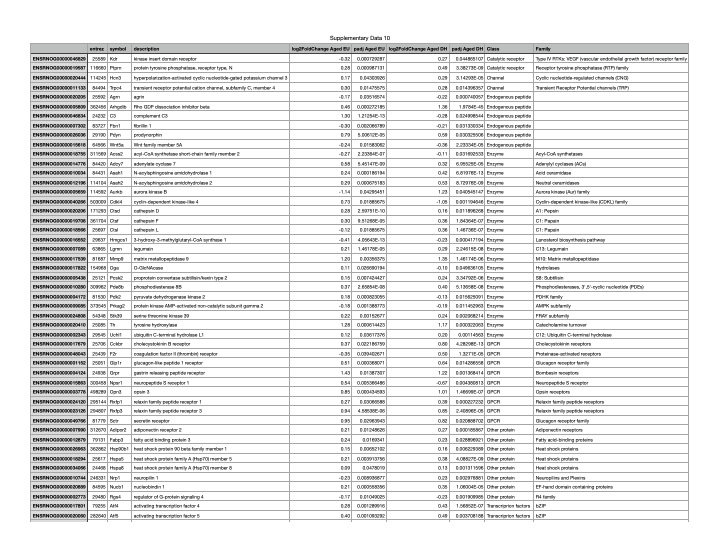

Supplement: Supplementary file 12 — Supplementary Data set 10 [file 41514_2023_108_MOESM12_ESM.zip › preview.jpg]

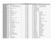

Supplement: Supplementary file 12 — Supplementary Data set 10 [file 41514_2023_108_MOESM12_ESM.zip › preview-micro.jpg]

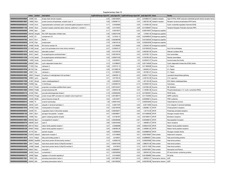

Supplement: Supplementary file 12 — Supplementary Data set 10 [file 41514_2023_108_MOESM12_ESM.zip › preview-web.jpg]

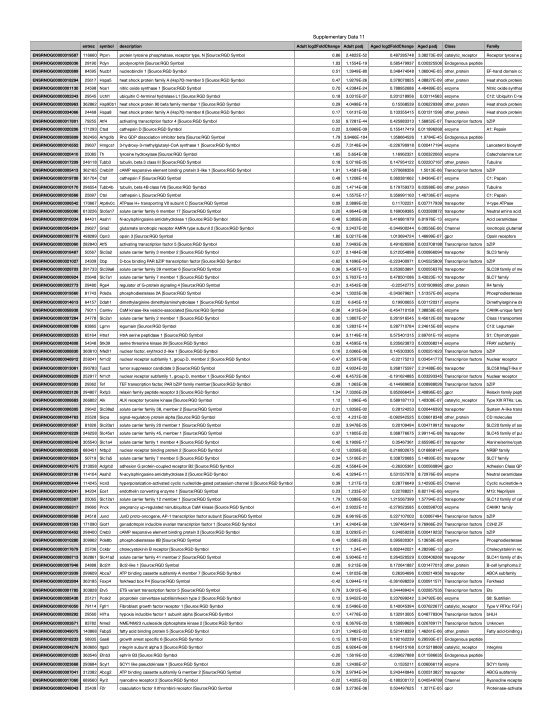

Supplement: Supplementary file 13 — Supplementary Data set 11 [file 41514_2023_108_MOESM13_ESM.zip › preview.jpg]

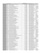

Supplement: Supplementary file 13 — Supplementary Data set 11 [file 41514_2023_108_MOESM13_ESM.zip › preview-micro.jpg]

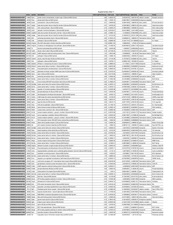

Supplement: Supplementary file 13 — Supplementary Data set 11 [file 41514_2023_108_MOESM13_ESM.zip › preview-web.jpg]

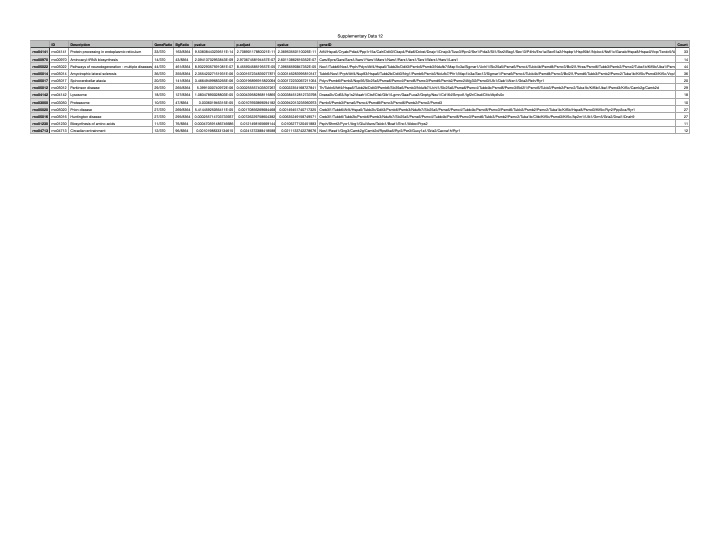

Supplement: Supplementary file 14 — Supplementary Data set 12 [file 41514_2023_108_MOESM14_ESM.zip › preview.jpg]

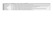

Supplement: Supplementary file 14 — Supplementary Data set 12 [file 41514_2023_108_MOESM14_ESM.zip › preview-micro.jpg]

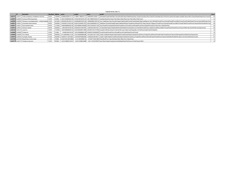

Supplement: Supplementary file 14 — Supplementary Data set 12 [file 41514_2023_108_MOESM14_ESM.zip › preview-web.jpg]

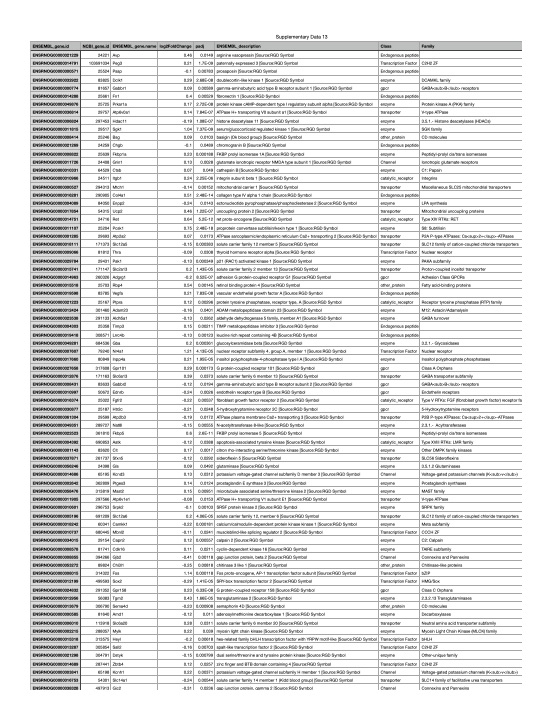

Supplement: Supplementary file 15 — Supplementary Data set 13 [file 41514_2023_108_MOESM15_ESM.zip › preview.jpg]

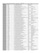

Supplement: Supplementary file 15 — Supplementary Data set 13 [file 41514_2023_108_MOESM15_ESM.zip › preview-micro.jpg]

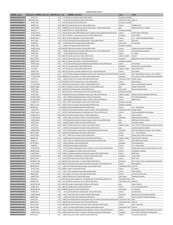

Supplement: Supplementary file 15 — Supplementary Data set 13 [file 41514_2023_108_MOESM15_ESM.zip › preview-web.jpg]

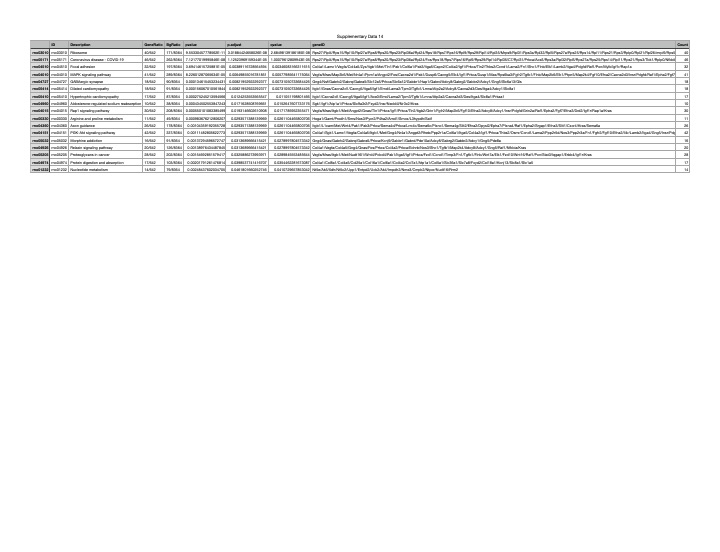

Supplement: Supplementary file 16 — Supplementary Data set 14 [file 41514_2023_108_MOESM16_ESM.zip › preview.jpg]

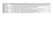

Supplement: Supplementary file 16 — Supplementary Data set 14 [file 41514_2023_108_MOESM16_ESM.zip › preview-micro.jpg]

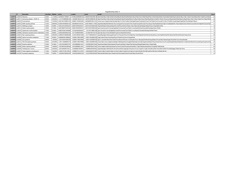

Supplement: Supplementary file 16 — Supplementary Data set 14 [file 41514_2023_108_MOESM16_ESM.zip › preview-web.jpg]

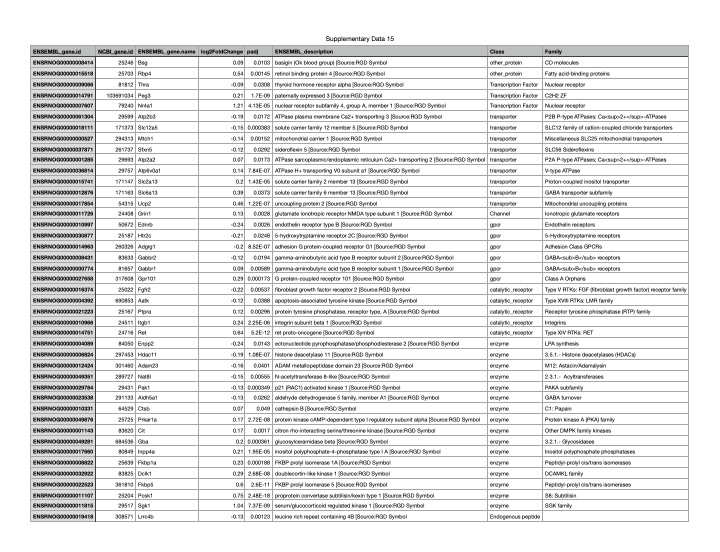

Supplement: Supplementary file 17 — Supplementary Data set 15 [file 41514_2023_108_MOESM17_ESM.zip › preview.jpg]

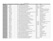

Supplement: Supplementary file 17 — Supplementary Data set 15 [file 41514_2023_108_MOESM17_ESM.zip › preview-micro.jpg]

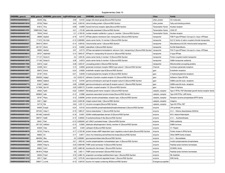

Supplement: Supplementary file 17 — Supplementary Data set 15 [file 41514_2023_108_MOESM17_ESM.zip › preview-web.jpg]

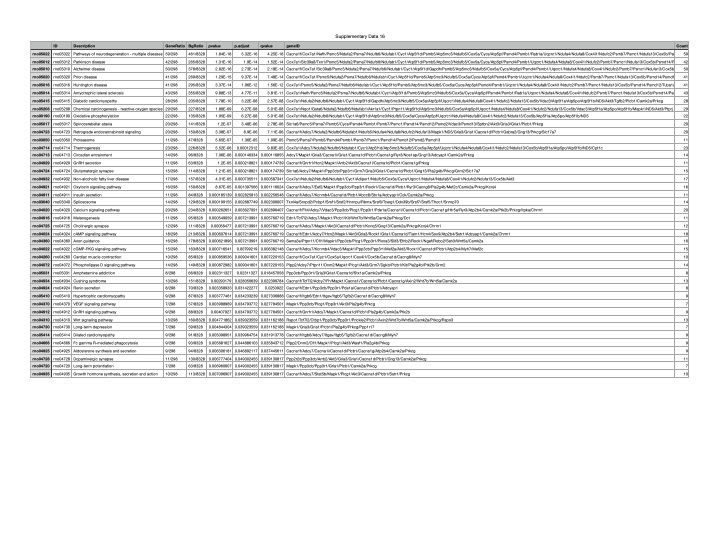

Supplement: Supplementary file 18 — Supplementary Data set 16 [file 41514_2023_108_MOESM18_ESM.zip › preview.jpg]

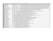

Supplement: Supplementary file 18 — Supplementary Data set 16 [file 41514_2023_108_MOESM18_ESM.zip › preview-micro.jpg]

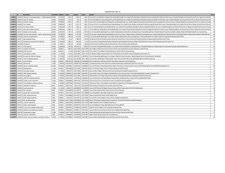

Supplement: Supplementary file 18 — Supplementary Data set 16 [file 41514_2023_108_MOESM18_ESM.zip › preview-web.jpg]
